# Selective Extraction Process of Niobium and Tantalum from Tin Slag Derived from the Thermal Processing of Cassiterite

**DOI:** 10.1021/acsomega.6c01589

**Published:** 2026-07-08

**Authors:** Darwin Michell Cheje Machaca, Rosario Belen Juyo Salazar, Susan Sofia Flores-Calla, Amilton Barbosa Botelho Junior, Denise Crocce Romano Espinosa, Jorge Alberto Soares Tenório

**Affiliations:** † Department of Chemical Engineering, Polytechnical School, University of Sao Paulo, São Paulo, SP 05508-080, Brazil; ‡ Universidad Nacional de San Agustín de Arequipa, Arequipa 04000, Peru; § Catholic University of Santa Maria, Arequipa 04013, Peru; ∥ Department of Chemical Engineering, Norwegian University of Science and Technology, Trondheim 7491, Norway

## Abstract

The metallurgical industry for niobium and tantalum extraction from secondary resources typically relies on conventional hydrometallurgical processes, based on acid leaching with hydrofluoric acid. However, these methods pose significant challenges due to the generation of nonselective solutions containing large amounts of codissolved impurities, as well as handling and safety risks in operational practices. In this context, the development of economically viable and environmentally friendly alternatives is essential. This study proposes an innovative and fluoride-free methodology for recovering Nb and Ta from tin slag. The material was subjected to thermal treatment with KHSO_4_, with operational parameters defined through thermodynamic simulations. The optimal conditions identified were a temperature of 600 °C, a slag-to-KHSO_4_ ratio of 1:3 (w/w), a particle size of −74 μm, and a calcination time of 3 h. Then, the material was leached with oxalic acid under the following conditions: a solid-to-liquid ratio of 33 g/L, a concentration of 0.5 M, a temperature of 60 °C, and a leaching time of 60 min. This approach achieved recovery efficiencies of 82% for Nb and 80% for Ta. Under optimal leaching conditions, the dissolution of impurities remained low, with approximately 4% U, 2% Th, and less than 1% Zr, while these elements predominantly remained in the solid residue. These results demonstrate the effectiveness and selectivity of the method, enabling the production of solutions suitable for subsequent purification stages.

## Graphical abstract

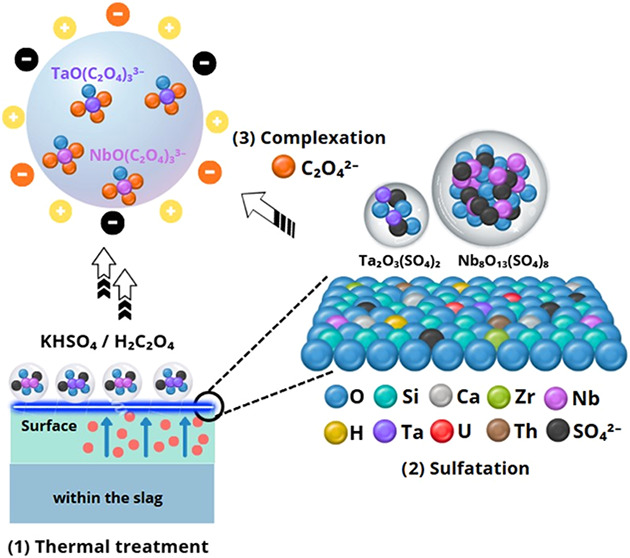

## 1 Introduction

The utilization of industrial waste is a major problem worldwide and the management of this waste is a prime challenge, especially when land is scarce. For instance, tin slag is a nonferrous byproduct of the cassiterite refining process.^1^ In past decades, tin slags were supply materials for Ta, contributing 50% of the world production in the 1980s and 1990s.^2^ Today, the production of this resource has gradually decreased over the last 20 years, with only a 4% increase forecasted for 2026, with fluctuating production values between 100,000 tons and 200,000 tons.^3^ Despite the statistical data, tin slags continue to persist as challenging resources for the recovery of metallic phases.

The composition of cassiterite (the main tin mineral) depends on its geolocation in different types of deposits; it is generally associated with complex polymetallic sulfide minerals, zircon, columbite, monazite, and silicate–carbonate gangue,^4,5^ containing in its crystal lattice metals such as Fe, Nb, Ta, Ti, Mn, and Al in the form of FeO–Fe_2_O_3_–Al_2_O_3_–CaO–SiO_2_, CaO–Ta_2_O_5_–SiO_2_, and CaO–Nb_2_O_5_–SiO_2_.^6–8^ Due to the stable chemistry of cassiterite, it rarely reacts with mineral acids or bases under common conditions; therefore, traditional metallurgical processes opt for flotation stages to produce high-quality concentrates associated with metallic oxides and gangue phases.^9^

The concentrates produced are sent to a smelting stage in blast furnaces with solid reducers (anthracite, bitumen, coke) and fluxes (limestone and quartz).^9^ In the first smelting stage, a tin-rich alloy and a ferrous-silicate slag with small amounts of lime and unreduced tin are produced, which are subsequently reintroduced into the circuit.^10,11^ In the second smelting stage, larger amounts of reagents (limestone, coal, and iron scrap) are used to facilitate the reduction of tin from SnO_2_ in the first slag and form FeSn alloys to be recirculated into the process, while the slag produced can be recirculated to a third smelting stage if its Sn content is recoverable or discarded through physical processes.^10,11^ The slags produced in the first, second, and final stages (Table 1) are vitreous in nature and contain varying amounts of lime, iron, silica, and metallic oxides within their interatomic networks, as a result of the addition of fluxes and the association of mineralogical phases adhered to cassiterite

**Table 1:** Composition of Slags at Different Stages of the Cassiterite Refining Process^11^ ^a^

	wt (%)					
Stage	SiO_2_	FeO	CaO	SnO_2_	Al_2_O_3_	MgO
first slag	30–40	15–25	5–15	5–25	NA	NA
second slag	24–28	12–20	20–22	NA	9–11	2–6
ground final slag	12–41	0.9–27.7	11–29	0.1–2.8	4.7–13	2–16

a NA = Information not available. Data compiled from ref 11.

It has also been reported that during the production stage, these slags contain economically elements such as Zr, Hf, Ti, U, Th, Nb, and Ta within the atomic lattices of the different material matrices.^1,11,12^ Among these elements, niobium and tantalum are particularly significant due to their refractory nature and intrinsic physicochemical properties, which make them indispensable for high-tech applications in industries such as aerospace, automotive, construction, nuclear energy, and microelectronics.^13^ These metals frequently occur together in nature within various resources, combined with oxide impurities, posing a challenge for their extraction and separation.

The traditional processing for extracting Nb and Ta consists of sequential steps based on hydrometallurgical and pyrometallurgical routes.^14^ The commercial-scale extraction approach focuses on operability with fluoride ions or their derivatives, but such processes have been hindered by operational and environmental challenges. It is well documented in the literature that the use of HF during leaching stages results in volatilization losses of 6–10% by volume. Additionally, due to the solvent action of the acid, large volumes of residues containing fluoride ions are generated, leading to increased costs for the treatment and regeneration of fluoride salts. This has been the primary focus of many research studies.^15–17^ However, another aspect that is not widely addressed is the presence of radionuclides (Th and U) during process operations, leading to more frequent challenges in handling, which must comply with state regulations during operational practices.^18^ These radionuclides generally occupy the interstitial spaces in the slag matrix, replacing Nb and Ta atoms in the crystalline lattice, posing a challenge for separation by physical methods.

In the face of this challenge, the literature has minimally documented that practical methods for the removal of Th and U from matrices are based on sulfation methods using H_2_SO_4_ solutions,^19^ or hot HF-H_2_SO_4_ systems HF–H_2_SO_4_.^20^ However, these methods may require variable temperature and concentration conditions, which can lead to partial or total dissolution of the metals of interest along with large amounts of coadjacent material being dissolved. These aspects reflect the limitation of using sulfuric acid as a single agent for the selective extraction of radionuclides from the target metals.

As an alternative to this method, roasting with potassium bisulfate salts has stood out for its extraction efficiency exceeding 80% of radionuclides.^21,22^ It is known that the decomposition mechanism begins at 300 °C eqs 1 and 2, generating highly oxidizing intermediate products capable of reacting with the metals present in the material matrices.^23^

1 $$2{\mathrm{KHSO}}_{4}\to 2K^{+}+2{\mathrm{HSO}}_{4}^{-}$$

2 $$2{\mathrm{HSO}}_{4}^{-}\to S_{2}O_{7}^{2-}+H_{2}O$$

Equation 2 shows that bisulfate decomposition occurs between 500–700 °C and forms pyrosulfate, which reacts with the metals in the matrix. For example, during the reaction phase of pyrosulfate with (Nb,Ta)_2_O_5_, oxygen atoms are replaced by sulfate groups, which allows overcoming the Nb/Ta–O bond energy barrier, leading to the formation of sulfated compounds soluble in aqueous media.^24^ Due to the complexity of the sulfation process, the phases formed during the process may lead to mixed sulfates and oxosulfates of niobium and tantalum, whose composition can vary significantly depending on the reaction conditions. Although the exact structure of the sulfated species is not fully established, it has been reported that the interaction of potassium ions from the reagent may be incorporated into the sulfate lattice, leading to the formation of complex phases of the type K–Nb–(SO_4_) and K–Ta–(SO_4_) with variable stoichiometry.^25,26^

However, despite this method having reported considerable leaching efficiencies for the radionuclides,^21,22^ and target metals.^23^ This reagent as a single agent lacks intrinsic activity for the efficient separation of the target metals and may be limited by other factors, such as high temperatures (>500 °C) and considerable stoichiometric amounts (>1:3 w/w),^23^ which may lead to reactivity similar to that of sulfuric acid. Therefore, there is a need to promote the exploration of complementary reagents capable of achieving efficient separation of the target metals, while simultaneously valorizing supply chains derived from secondary resources for industrial utilization.

Oxalic acid is one of the most promising organic acids acting as leaching and reducing reagents. Its application has been extensively carried out for the extraction of metals such as Fe, Ti, V, W, etc.^27–30^ as well as in gangue waste separation processes in minerals or secondary resources.^29,31^ This reagent exhibits better versatility and environmental sustainability; although its acidity is weak compared to that of sulfuric acid, its efficiency is comparable to sulfuric acid at the same concentration.^32^ To efficiently leach metals and achieve the economic and environmental objectives of the industry, it is necessary to use a combination of roasting with an organic acid as the leaching solvent. For example, Conrado Da Luz demonstrated a fluoride-ion-free methodology through alkaline roasting followed by leaching with H_2_C_2_O_4_ (1 M; S/L 1:30; 55 °C, 240 rpm for 3 h), achieving 97% Nb and 76% Ta leaching. Meanwhile, for Th, U, and other elements, the reported values did not exceed 60%.^33^ Shikika et al.^31^ carried out a combined process of alkaline roasting, precipitation, and redissolution with oxalic acid, showing efficiencies of 92% Nb and 86% Ta. Machaca et al.^24^ reported a combined thermal treatment of H_2_SO_4_–KHSO_4_, followed by oxalic leaching, obtaining a liquor containing 94% Nb and 75% Ta, while data for the other elements (Th + U) are not available in the document. However, to date, it is known that there is virtually no systematic experimental study, and the leaching of Nb/Ta and the simultaneous separation of radionuclides from spent tin slags using a combination of roasting and oxalic acid as the leaching agent has rarely been reported. In addition, greater attention should be paid to the mechanism of bisulfate roasting and oxalic acid leaching, as it plays an important role in enhancing the leaching rate of the target metals from secondary wastes such as tin slags.

In this context, this study investigates a fluoride-ion-free methodology for the efficient and selective extraction of Nb and Ta from tin slags, using thermal treatment based on thermodynamic modeling with potassium bisulfate and oxalic acid leaching. The process is optimized to reduce impurities and minimize the presence of radionuclides such as Th and U. The proposed approach emphasizes high selectivity, limiting the codissolution of these elements compared to conventional methods, and enabling safer and more sustainable processing. Our study contributes to the development of sustainable mining practices.^34^

## 2 Materials and Methods

### 2.1 Raw Material and Chemical Products

A Brazilian company supplied approximately 120 kg of slag from the cassiterite reduction process.^31^ The particle size of the analyzed slag was 0.811 mm, as shown in Figure S1 (Supporting Information), using a set of sieves arranged in a mechanical shaker (GLASSLAB 12499). For this study, analytical grade reagents were used, which included potassium bisulfate (KHSO_4_) in the thermal treatments, while oxalic acid (H_2_C_2_O_4_), citric acid (H_6_C_4_O_5_), tartaric acid (H_6_C_4_O_6_), ascorbic acid (H_8_C_6_O_6_), and ethylenediaminetetraacetic acid (EDTA) were used for the leaching tests. Before use, all these reagents were dried at 105 °C for at least 24 h to remove moisture, and the solutions were prepared with Milli-Q water (<0.05 μS·cm^–1^).

### 2.2 Characterization of Tin Slag

The slag composition was determined using inductively coupled plasma optical emission spectrometry (ICP-OES, Agilent 710), energy-dispersive X-ray fluorescence (EDXRF, PANalytical Epsilon 3XL), AAS (Shimadzu AA-7000) and wavelength dispersive X-ray fluorescence (WEDXRF). The phases present in the material were identified by X-ray diffraction (XRD, Rigaku MiniFlex300, Cu–Kα, λ = 1.54 Å). The slag’s morphology and quantitative composition were analyzed using scanning electron microscopy with EDS (SEM-EDS, Phenom ProX, 15 kV). Samples from the thermal treatment and leaching experiments were analyzed by EDXRF to quantify Nb, Ta, U, Th, and Zr.

### 2.3 Methodology

#### 2.3.1 Thermodynamic Simulation

The thermodynamic behavior of tin slag was evaluated using the FactSage v8.3 software (GTT Technologies, Aachen, Germany, and Thermfact/CRCT, Montreal, QC, Canada), with the integrated thermodynamic databases FactPS and FToxid, which include properties of solid, liquid, and gaseous phases.^35^ The software enabled the prediction of phase equilibria and chemical reactions under experimental temperature conditions (25–1000 °C) and stoichiometric ratios of the Slag-KHSO_4_ system with molar ratios of 1:2, 1:4, and 1:6 (mol:mol), minimizing the Gibbs free energy for the input compositions.

#### 2.3.2 Experimental Procedure

The integrated roasting–leaching process is illustrated in Figure 1. First, the initial parameters for the thermal treatment were defined based on the thermodynamic simulation. Although the thermal modeling does not consider the optimal particle size, the material was comminuted in a HEZROG device until obtaining a fine particle size of less than 75 μm, where a higher degree of metal liberation occurs.^36^ The comminuted material was analyzed using the MALVERN (MasterSizer 2000) instrument. Therefore, for the thermal treatment tests, 5 g of the comminuted material with a particle size of 70 μm (Supporting Information, Figure S2) were used, which were uniformly mixed with KHSO_4_ at a ratio of 1:3 (w/w). The mixture was then homogenized and placed in a muffle furnace (JUNG, LF00612), where it was roasted at a temperature of 600 °C for a fixed time of 3 h.

**Figure 1:**
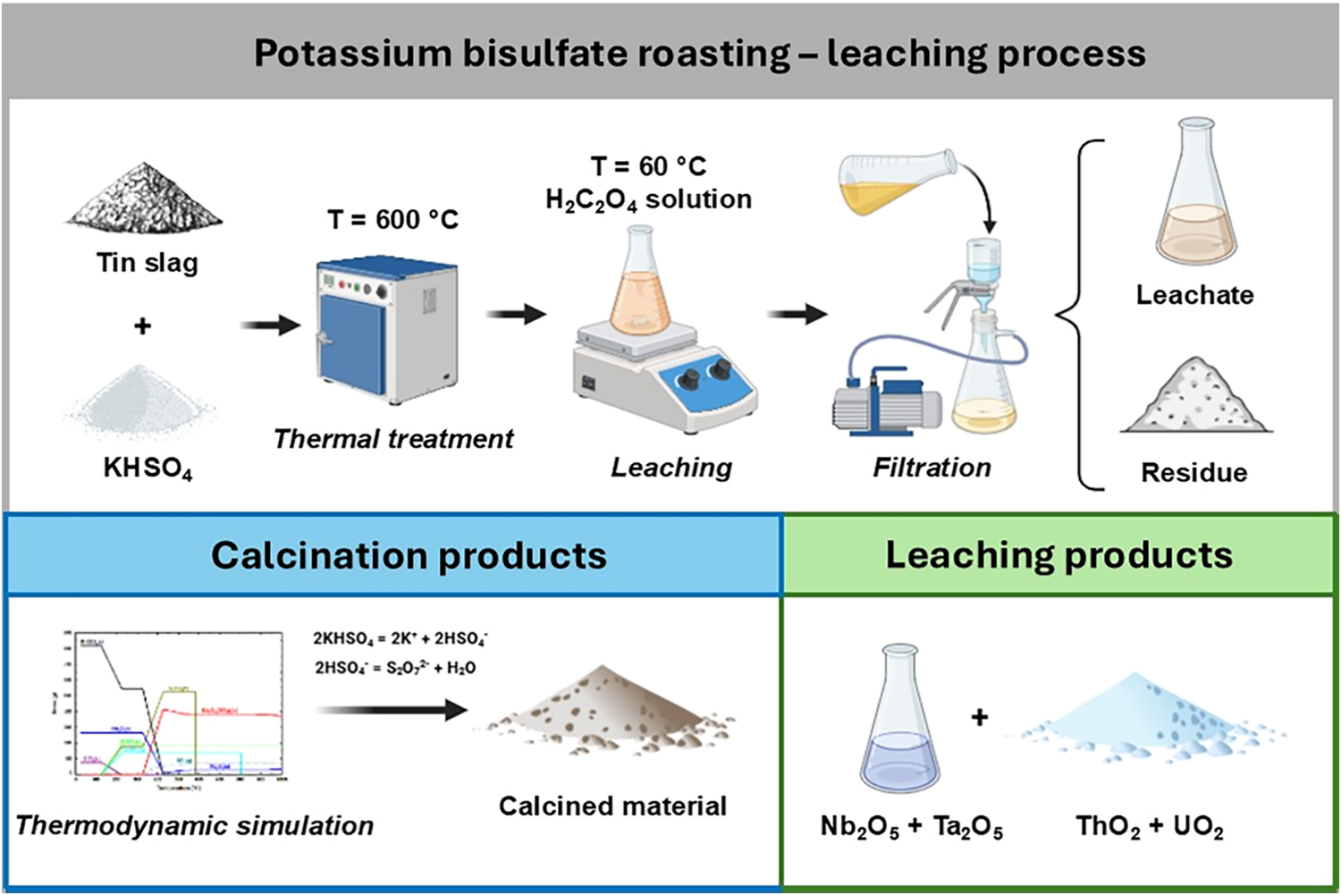
Process flow diagram for the selective extraction of niobium and tantalum from tin slag.

The material obtained after the thermal treatment tests was subjected to an experimental leaching procedure with oxalic acid under constant agitation and controlled conditions of S/L ratio, concentration, temperature, and time in a glass reactor equipped with a condenser. Table 2 presents the experimental conditions evaluated in the leaching tests.

**Table 2:** Evaluation of Leaching Conditions

Parameters	Conditions
Solid–Liquid ratio (g L^–1^)	20, 25, 33, 40, 50 and, 100
Concentration (mol L^–1^)	0.1, 0.25, 0.5, 0.75, 1, and 2
Temperature (°C)	25, 40, 60, 70, 80, and 90
Time (min)	60, 90, 120, 150, and 180

After the leaching tests, the samples were cooled to room temperature and filtered. The resulting leachates were analyzed by EDXRF in duplicate, with the average of the results being used. The solid residues were washed with deionized water and filtered using a vacuum pump. The residue was dried in an oven at 60 °C for 24 h for characterization by XRD and WEDXRF.

Following the determination of the optimal leaching parameters using H_2_C_2_O_4_, a comparative experiment was conducted employing the same parameters to evaluate the efficiency of H_2_C_2_O_4_ relative to other organic acids such as H_6_C_4_O_5_, H_6_C_4_O_6_, H_8_C_6_O_6_, and EDTA.

The leaching rate (η) of all the evaluated elements was calculated according to eq 3

3 $$\eta =\frac{c\times V}{m\times w}$$

Where *c* is the concentration of the leached element (wt %); *V* is the volume of the leachate (mL); *m* is the mass of slag used (g); and *w* is the content of the corresponding element in the slag (wt%).

## 3 Results and Discussion

### 3.1 Characterization

The chemical analysis of the slag by AAS, EDXRF, and ICP-OES is shown in Figure 2. The sample is primarily composed of 35.5% SiO_2_, 18% CaO, and 15.5% ZrO_2_. These results were to be expected, since during the cassiterite refining stages, gangue residues such as silica (12–41% SiO_2_) and added additives such as calcium salts decompose at temperatures >1000 °C, appearing as oxides (11–29% CaO) in the final slag structure.^11^ In addition, the presence of minerals associated with cassiterite, such as zircon and columbite common in Brazilian soils,^10^ indicates the high concentration of ZrO_2_, together with niobium and tantalum oxides, which typically vary between 1–25%,^16^ (U, Th)­O_2_ between 0.2–2%,^33,37^ and other minor constituents representing 1.8% (Zn 0.6%, Mn 0.6%, Y 0.2%, In 0.2%, Ba 0.2%).

**Figure 2:**
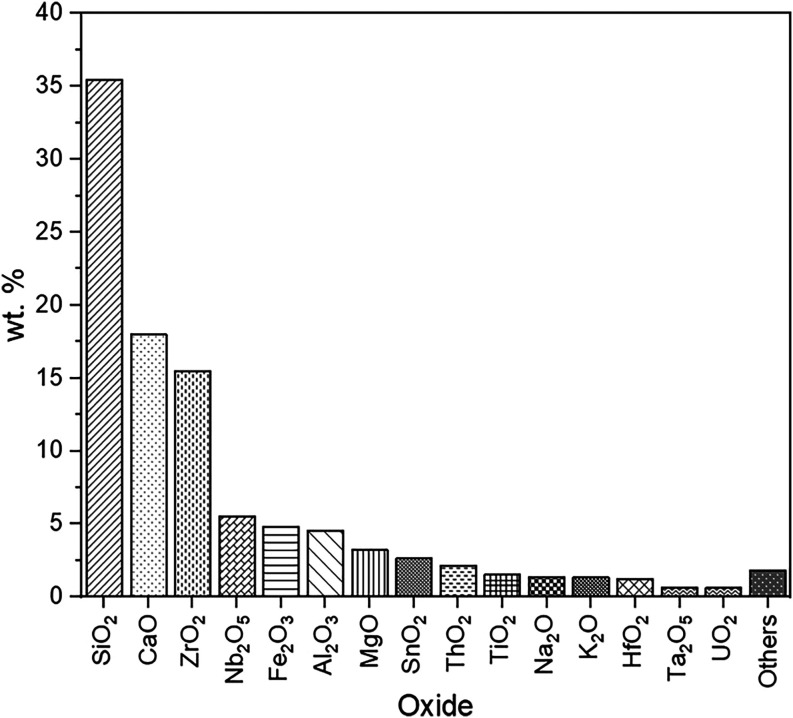
Characterization of tin slag.

The refractory nature of tin slag is complex, and most of the elements determined by elemental analysis are believed to be present in the matrix as oxidized phases. In tin slags, their mineralogical representation is based on ternary CaO–SiO_2_–FeO systems,^38^ but for Brazilian slags, due to their low iron content and the predominance of major components such as ZrO_2_, their representation is based on CaO–SiO_2_–ZrO_2_ systems, as has already been proposed by different authors.^10,24,37^ However, to date, no detailed study exists regarding their chemical–surface morphology, and it is only known from the TIMA–MIRA analysis system reported in the work of Clemente et al.^10^ that high–electron–density metallic elements are dispersed in the slags within silicate phases and mainly in zircon, baddeleyite, and augite phases. This elemental distribution has also been addressed by Fan et al.,^36^ Ma et al.,^39^ and Huang et al.,^40^ that revealed through analytical techniques such as X–ray photoelectron spectroscopy (XPS), Bulk Process Mineralogy Analysis (BPMA), and quantitative evaluation of materials by scanning electron microscopy (QEMSCAN), that the elements in slag residues are associated with extremely refractory phases (Ca_3_ZrSi_2_O_9_, Ca_2_ZrSi_4_O_12_, CaSiO_3_) and that their ionic form (M^+^) in the matrix increases when particle size fractions are fine (−100 μm). In this context, the XRD and SEM–EDS analyses of the tin slag show a correlation in its representation as oxidized phases and in the distribution of the elements within the highly refractory matrix, which explains its limited dissolution in the presence of mineral acids and validates the proposed mechanism of action.

The mineralogical analysis was carried out by X-ray diffraction using the Rigaku PDLX 2 software database, which incorporates the ICDD PDF-4+ database (version available in the licensed system) and the Crystallography Open Database (COD), including entry No. 9012043 for phase identification. The main phases identified are shown in Figure 3, which reveals characteristic phases of SiO_2_ (PDF 00–076–0936), CaSiO_3_ (PDF 00–072–2297), and ZrO_2_ (PDF 00–083–0941). The identified ZrO_2_ is an intrinsic characteristic of Brazilian soils.^10^ Meanwhile, the SiO_2_ and CaSiO_3_ phases stem from the pyrometallurgical process of cassiterite.^41^ In the analysis, Nb and Ta phases remained below the detection threshold of the applied analytical technique. According to Conrado Da Luz and Oliveira reported that Nb and Ta phases were not identified in the slag matrix because their mass concentration was lower than 5%.^33,37^

**Figure 3:**
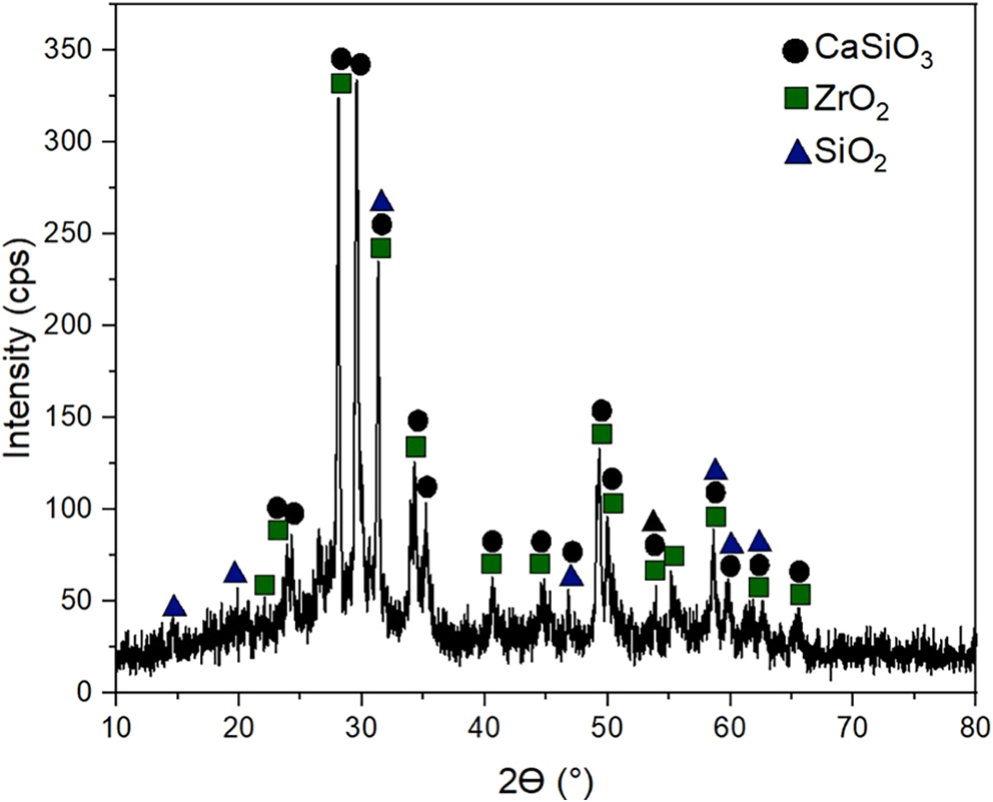
XRD pattern of the tin slag.

Figure 4 shows the SEM-EDS images of the slag, highlighting the heterogeneity of the particles. The EDS spectrum reveals that all irregular particles in the matrix are encapsulated by the glassy silicate phase, which appears in a grayscale with varying intensities. The EDS spectrum of the selected point (#1) in the image shows an irregular gray particle with higher intensity, mainly attributed to a mixture of calcium and silicon oxides in the form of silicates, originated by fusion during the smelting stages.^24,37^ In addition, small metallic fractions (Al, Ti, Nb, Mg, Fe, and K) encapsulated in the amorphous silicate phase are observed. Likewise, at point (#2), it is observed that the selected regular particle presents inclusions of higher metallic density visible within the gray regions, which are attributed to the metallic presence of major components such as zirconium oxide, resulting from its adhesion to the base mineral, together with metallic fractions such as Sn, Ti, Nb, Ta, Al, and Fe, in accordance with the EDS and XRD analyses.

**Figure 4:**
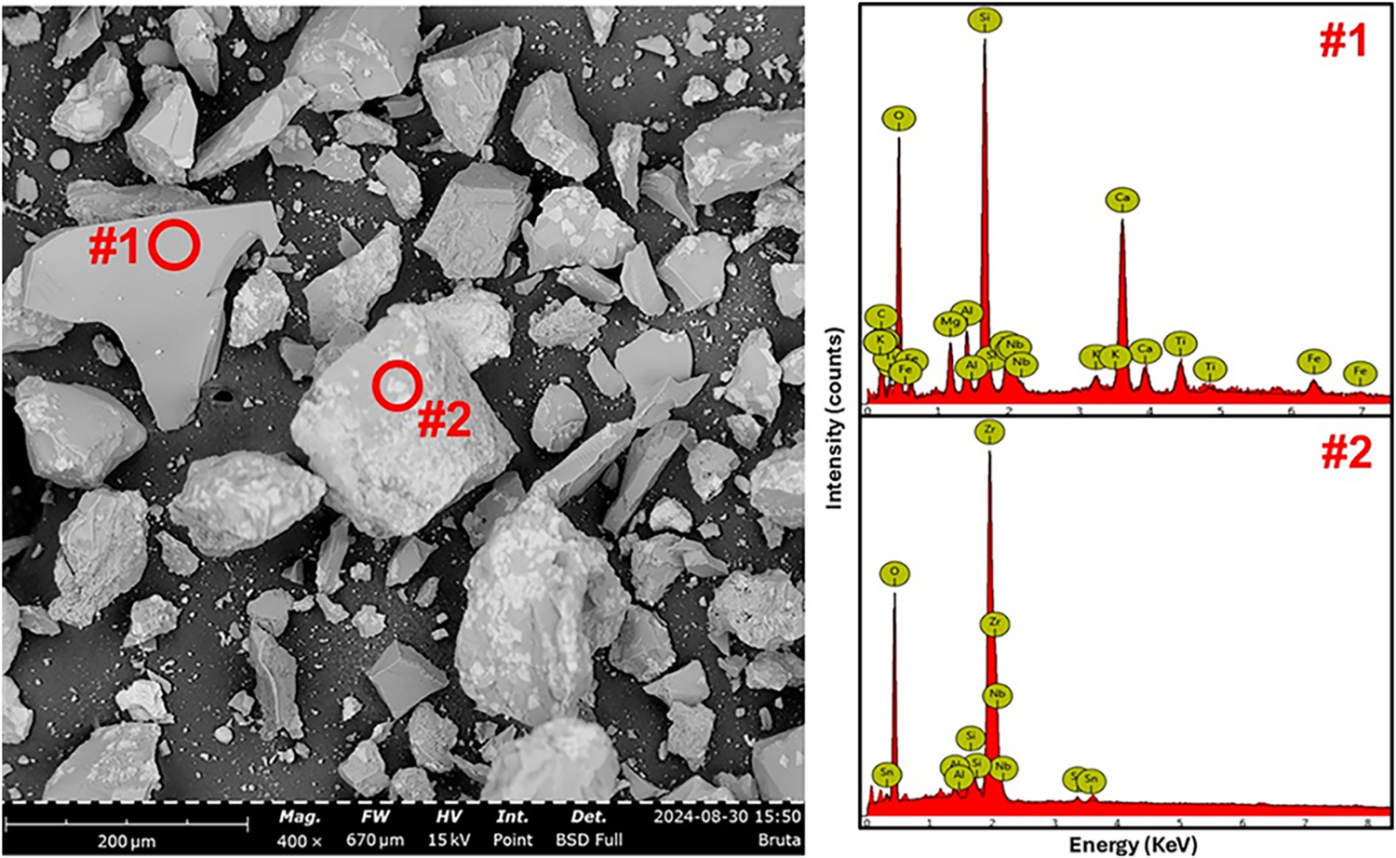
SEM-EDS analysis of the tin slag.

### 3.2 Thermodynamic Simulation

The residual matrices are composed of impurities or elements present as metallic oxides, propitiating unwanted secondary reactions during the thermal treatment stage.

Figure 5 illustrates the chemical behavior of the Slag–KHSO_4_ system at different stoichiometric mass ratios, focusing on sulfation reactions. Due to the limited thermodynamic data for tantalum, the simulations were based on niobium. In thermodynamic modeling, it can be observed that the decomposition reaction of KHSO_4_ begins at 125 °C, but its complete decomposition occurs starting at 350 °C, where metallic sulfation takes place, as previously reported in the literature.^23,42^ However, temperature is not the only critical factor in practice; the amount of reagent required to ensure product formation is also crucial.

**Figure 5:**
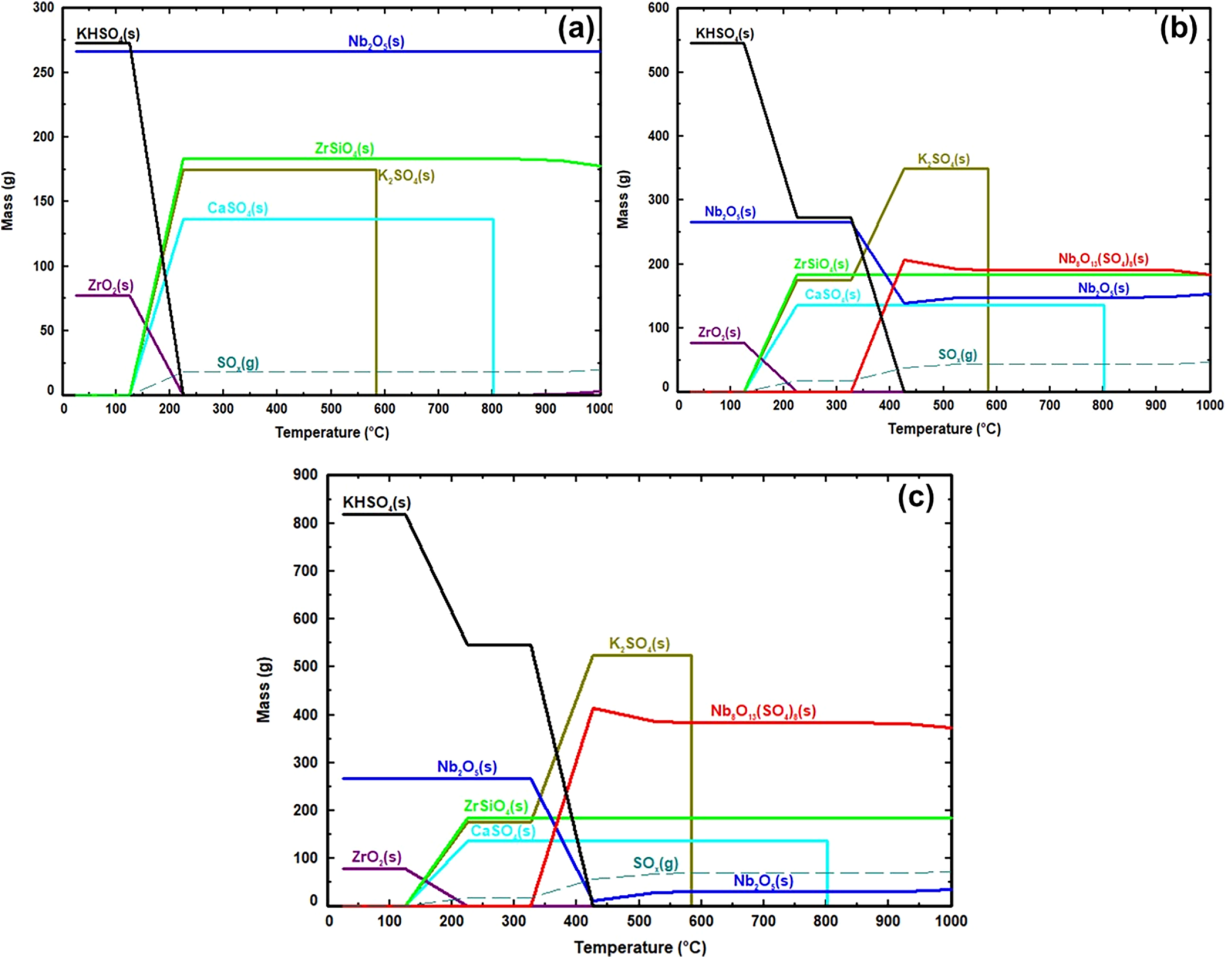
Thermodynamic simulation of the Slag–KHSO_4_ system: (a) 1:2 molar ratio, (b) 1:4, and (c) 1:6 (performed using Fact*Sage* v8.3).

Figure 5a shows that the mass of niobium (265.8 g, equivalent to 1 mol of Nb_2_O_5_) remains as unreacted Nb_2_O_5_ over the entire evaluated temperature range, evidencing the absence of sulfation under this condition. In contrast, from 200 °C onward, the formation of approximately 1 mol of CaSO_4_ (136.1 g) and 1 mol of K_2_SO_4_ (348.5 g) is observed, indicating that the sulfating agent preferentially reacts with the matrix components. Likewise, zirconium forms 1 mol (183.3 g) of the insoluble species ZrSiO_4_ from 200 °C, as a result of the interaction between the siliceous matrix and the reactive system. On the other hand, Figure 5b indicates that as the stoichiometric ratio of the reagent increases, the formation of approximately 2 mol of K_2_SO_4_ is favored, and partial sulfation of niobium is observed above 350 °C. This is reflected in a decrease of the Nb_2_O_5_ mass by approximately 100 g and in the formation of about 200 g of Nb_8_O_13_(SO_4_)_8_. However, in both cases, the limited stoichiometric availability of KHSO_4_ restricts the overall conversion of Nb_2_O_5_, evidencing that these conditions are not sufficient to achieve efficient sulfation of the target metal. Our hypothesis in this regard is the limited catalytic activity of KHSO_4_ to generate (HSO_4_^–^) ions required for the sulfation reaction, as shown in eq 4.^43^ According to Amer et al.^44^ and El-Hazek et al.,^45^ insufficient amounts of KHSO_4_ limit the catalytic activity of the reagent, impacting the metal recovery rates.

4 $$2{\mathrm{HSO}}_{4}^{-}\to H_{2}{\mathrm{SO}}_{4}+{\mathrm{SO}}_{4}^{2-}$$

In contrast, as the molar ratio increases to 1:6 (Figure 5), the sulfation reaction becomes significantly more pronounced above 400 °C, favoring the formation of approximately 3 mol of K_2_SO_4_ and a mass conversion of around 23% into Nb_8_O_13_(SO_4_)_8_. This transformation is accompanied by a marked reduction of approximately 95% in the unreacted Nb_2_O_5_ phase. These results demonstrate that an excess of KHSO_4_ is crucial to promote the formation of stable sulfated species and to improve the conversion of Nb_2_O_5_. Consequently, to maximize the leaching efficiencies of Nb and Ta, a molar ratio of 1:6 was selected, which translates to 1:3 (w/w) in terms of chemical units, with a temperature of 600 °C. The reaction time was set at 3 h, as previously reported in several studies on successful decomposition procedures using bisulfate fusion for Nb and Ta minerals.^23,42,44,45^

The possible reactions that occur during this bisulfate treatment stage are shown in reactions 5, 6, 7, 8, 9, and 10. HSC Chemistry 6.0 software was used to calculate the Gibbs free energy as a function of temperature, and the results were plotted in Figure 6. Reaction 5, 6, 7, 8, and 9 show the interaction of bisulfate with the predominant phases of the matrix to form sulfated components within the 0–1000 °C range, indicating that these reactions can occur spontaneously (Δ_r_ *G* ^Θ^ < 0). In particular, reaction 10 shows the sulfation of niobium, which presents very negative Δ*G* values that become increasingly favorable with temperature, confirming that the process is entropy-driven and favored at elevated temperatures. Based on this result, it is demonstrated that niobium sulfate can be preferentially formed during bisulfate roasting at molar ratios of 1:6 mol/mol. In contrast, reactions 7 and 9 shows that zirconium sulfation is nonspontaneous (0–650 °C) due to slow formation kinetics and becomes spontaneous when the temperature exceeds 650 °C, which explains why it is not experimentally sulfated. Reaction 8 is spontaneous and shows that zirconium remains in the refractory phase in the form of zircon, corroborating the information provided by reactions 7 and 9. These calculations indicate that the sulfation of calcium, potassium, and niobium is thermodynamically feasible, whereas the sulfation of zirconium shows a limited tendency in the process, as verified in Figure 6.

5 $$6{\mathrm{KHSO}}_{4}+3\mathrm{CaO}\to 3{\mathrm{CaSO}}_{4}+3K_{2}{\mathrm{SO}}_{4}+3{\mathrm{HO}}_{2}$$

**Figure 6:**
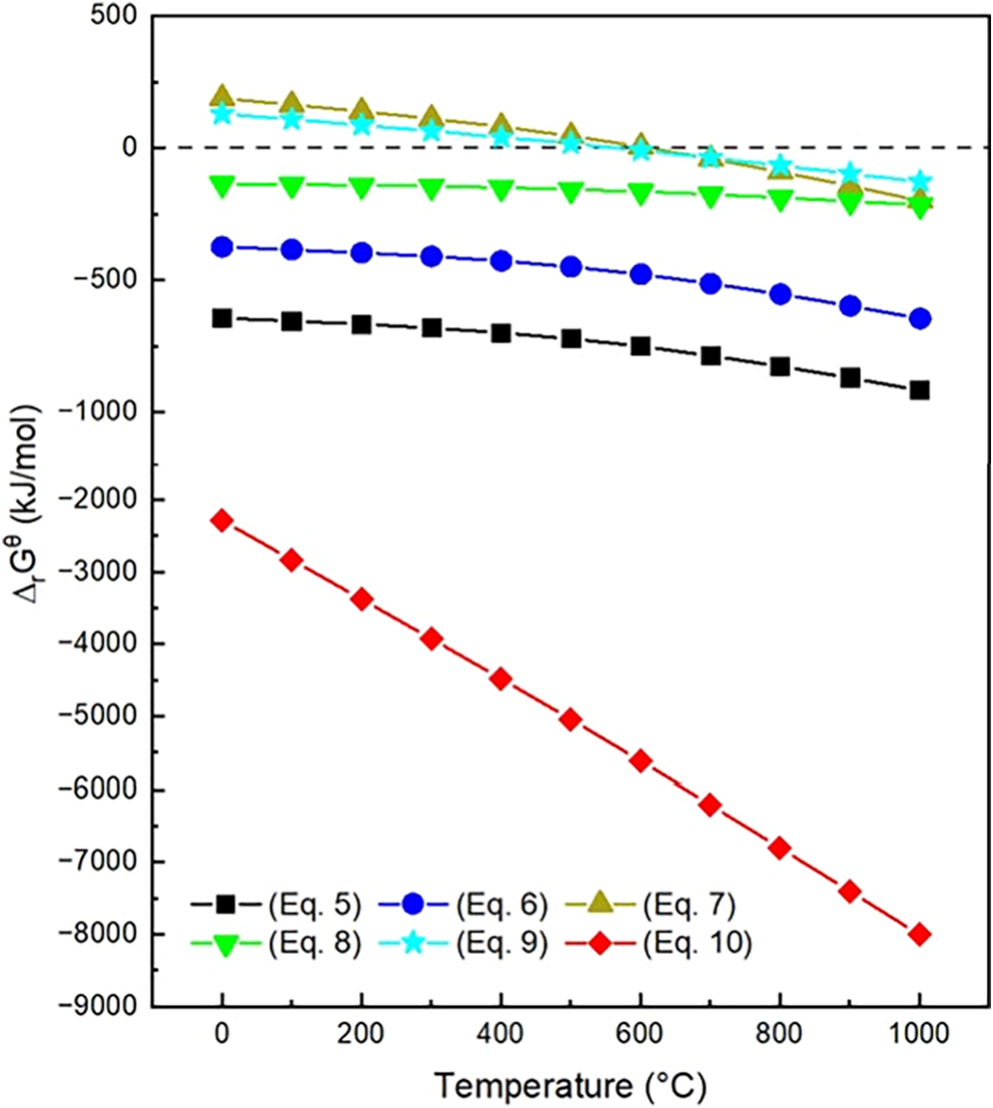
Correlation between Δ_r_ *G* ^Θ^ and temperature for eqs 5–10.


6 $$6{\mathrm{KHSO}}_{4}+3{\mathrm{CaSiO}}_{3}\to 3{\mathrm{CaSO}}_{4}+3K2{\mathrm{SO}}_{4}+3{\mathrm{SiO}}_{2}+3H_{2}O$$

7 $$6{\mathrm{KHSO}}_{4}+{\mathrm{ZrO}}_{2}\to \mathrm{Zr}({\mathrm{SO}}_{4}{)}_{2}+3K_{2}{\mathrm{SO}}_{4}+{\mathrm{SO}}_{3\left(g\right)}+3H_{2}O$$

8 $$2{\mathrm{KHSO}}_{4}+{\mathrm{ZrO}}_{2}+{\mathrm{CaSiO}}_{3}\to K_{2}{\mathrm{SO}}_{4}+{\mathrm{ZrSiO}}_{4}+{\mathrm{CaSO}}_{4}+H_{2}O$$

9 $$2{\mathrm{KHSO}}_{4}+{\mathrm{ZrO}}_{2}+{\mathrm{SiO}}_{2}\to {\mathrm{ZrSiO}}_{4}+K_{2}{\mathrm{SO}}_{4}+H_{2}O+{\mathrm{SO}}_{3\left(g\right)}$$

10 $$16{\mathrm{KHSO}}_{4}+4{\mathrm{Nb}}_{2}O_{5}+1/2O_{2\left(g\right)}\to {\mathrm{Nb}}_{8}O_{13}{\left({\mathrm{SO}}_{4}\right)}_{8}+8K_{2}{\mathrm{SO}}_{4}+8H_{2}O$$

### 3.3 Thermal Treatment

The thermal treatment of the slag with potassium bisulfate (KHSO_4_) was conducted at 600 °C, with a weight-to-weight ratio of 1:3, in a muffle furnace for 3 h. After the thermal process, the sample was characterized by XRD (Figure 7), and the data were processed with Rigaku PDXL 2 software, identifying phases of K_2_Ca_2_(SO_4_)_3_ (PDF 00–074–0327), K_7_Nb­(SO_4_)_6_ (PDF 00–079–1277), ZrO_2_ (PDF 00–072–0597), and SiO_2_ (PDF 00–081–0069) as the predominant phases. The results indicate the formation of sulfated phases, particularly K_7_Nb­(SO_4_)_6_, whose formation is favored under conditions of excess sulfating agent, highlighting the influence of reactant stoichiometry on the final composition of the obtained phases.^26^ These results are consistent with previous studies that report the effectiveness of bisulfate fusion for the treatment of materials containing niobium and tantalum.^23,42,44,45^

**Figure 7:**
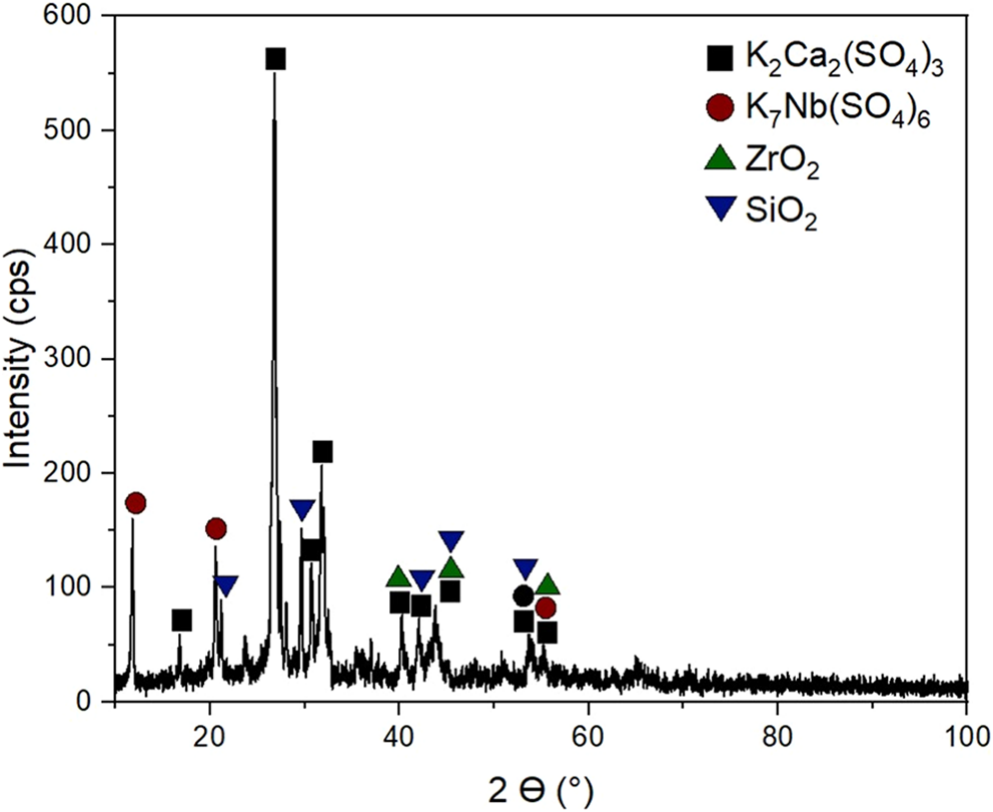
XRD pattern of the thermally treated material under the conditions: 600 °C, slag/KHSO_4_ ratio of 1:3 w/w for 3 h.

### 3.4 Leaching

After the thermal process, the feasibility of the proposed route was evaluated through oxalic acid leaching for the extraction of Nb and Ta. To understand the stability of the compounds formed during leaching, a chemical equilibrium diagram was generated using the HYDRAMEDUSA v.6 software, which allows the prediction of species solubility. Figure 8 shows the speciation diagrams of Nb, Th, U, and Zr at different oxalate concentrations as a function of pH, where Nb and Ta are considered the target elements, while Th, U, and Zr are treated as impurities.

**Figure 8:**
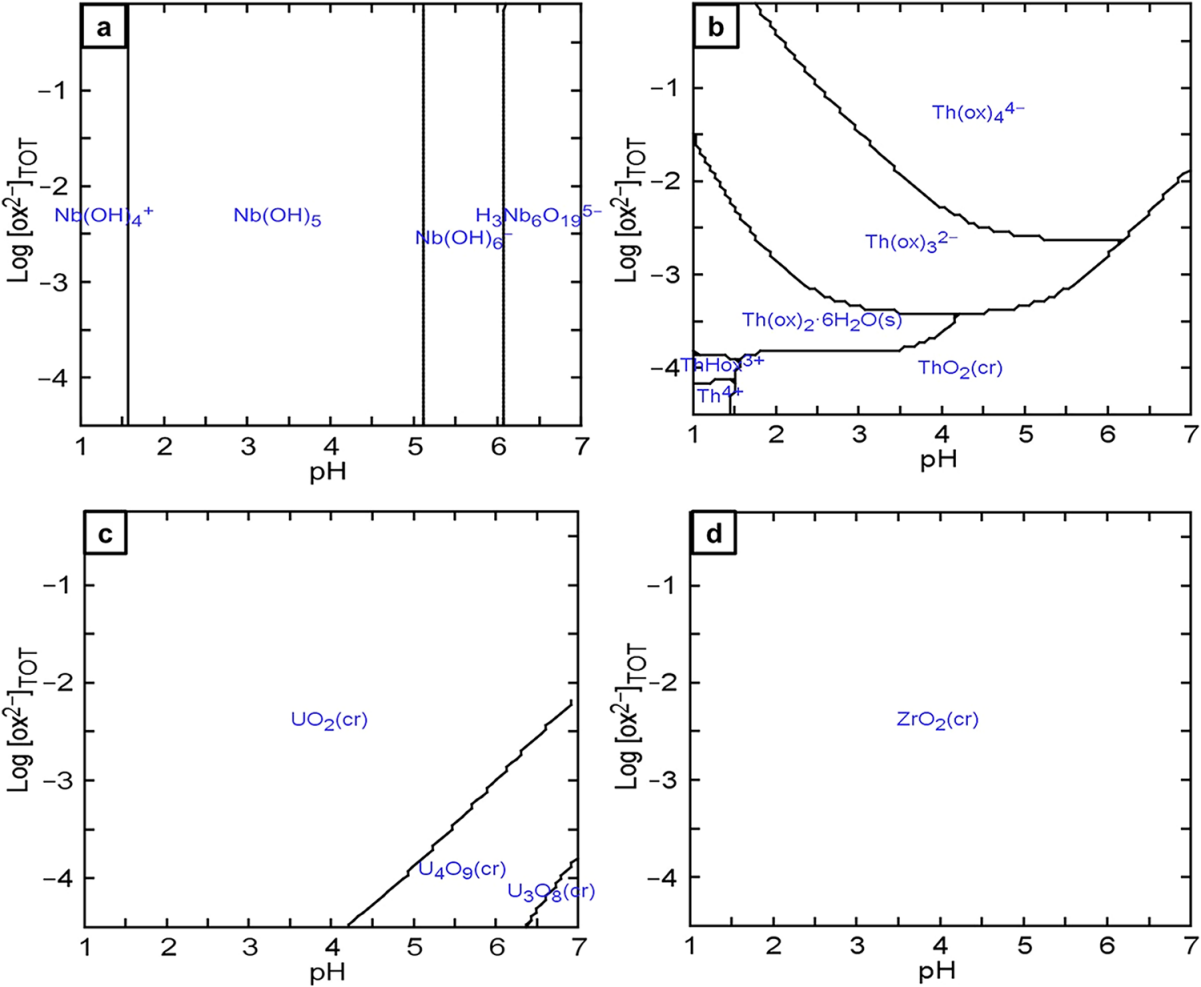
Predominance diagram of niobium (a), thorium (b), uranium (c), and zirconium (d) compounds evaluated as a function of pH and oxalate concentration. Conditions: [Nb^5+^] = 0.00066 mol, [Th^4+^] = 0.000128 mol, [U^4+^] = 0.000034 mol, and [Zr^4+^] = 0.00203 mol per liter of solution at 25 °C (based on the data from the initial characterization). The notation “ox^2–^” corresponds to the oxalate ion (C_2_O_4_^2–^).

The equilibrium diagram for Nb is shown in Figure 8a. It is observed that, in acidic media, sulfated niobium species can be transformed into hydrated forms such as Nb­(OH)_4_^+^ at low ligand concentrations,^46^ which subsequently form stable complexes such as [NbO­(C_2_O_4_)_3_]^3–^.^47^ This complexation process is well established in the literature and leads to the formation of highly soluble oxalate species at pH < 2.^24,31,47^ Although the equilibrium diagrams for Ta were not included due to limitations in the database,^48^ its behavior is expected to be similar to that of Nb due to their chemical similarity and proximity in the periodic table.

In contrast, the impurities (Th, U, and Zr) exhibit a different behavior under the same leaching conditions. Figure 8b shows the speciation diagram of Th. It can be observed that at concentrations (<0.003 M), insoluble species (Th­(ox)_2_·6H_2_O, ThO_2_), ionic species (Th^4+^, ThHOx^3+^), and oxalic species predominate depending on the pH of the solution. At concentrations higher than 0.003 M, a predominance of oxalic species is observed. However, it should be noted that the stabilization of Th­(ox)_3_^2–^ and Th­(ox)_4_^4–^ complexes at moderate pH with excess oxalate depends on specific pH and concentration conditions due to the possible formation of thorium oxalate precipitates (Th­(Ox)_2_·*n*H_2_O) or colloidal particles that are not transferred to the liquid phase. This has already been demonstrated by Erten et al.,^49^ that showed that the complexation constants are dependent on the ionic strength, so a high ionic strength in the medium may decrease the apparent effectiveness of the ligand. Figure 8c shows a reduced solubility of U when working at pH < 4 over a wide range of concentrations. This contrasts with what was reported by Lapidus and Doyle.^48^ who evidenced that uranium exhibits a wide solubility range, possibly due to the presence of phosphate compounds that shift the solubility behavior. On the other hand, Figure 8d shows that Zr does not react with oxalate across all the evaluated pH and concentration ranges. This is evident because, during sulfation, crystalline zirconia is predominantly formed, while only a small fraction is chelated with sulfate ions, which upon hydration with oxalate ions form zirconium hydroxo complexes that are insoluble under acidic pH ranges.^50^ Furthermore, the lack of thermodynamic data in the software limits the representation of such insoluble species.

Therefore, in order to maximize the extraction of Nb and Ta and minimize the dissolution of impurities (Th, U, and Zr), conditions were selected in which oxalate is present in excess while maintaining a strongly acidic environment (pH < 2). Under these conditions, the preferential formation of stable Nb and Ta oxalate complexes is favored, whereas the low solubility and precipitation tendencies of Th, U, and Zr limit their codissolution. Additionally, although these impurities may slightly influence the system through competition for oxalate ions or changes in ionic strength, their overall impact on Nb and Ta leaching is minimal. Consequently, after evaluating the presented Pourbaix diagrams and the corresponding literature, a solid-to-liquid ratio of 20 g/L, a reagent concentration of 0.5 M, a temperature of 60 °C, and a time of 180 min were selected as the starting condition. In order to improve the selectivity of the process, enabling a higher efficient extraction of Nb, while other elements such as Th, U, and Zr remain in the solid residue.^24,31,33^

#### 3.4.1 Effect of Solid–Liquid Ratio

After the thermal treatment with potassium bisulfate, Nb and Ta were recovered with 250 mL of the acidic solution and the thermally treated slag. The variation of the solid/liquid ratio was studied at 60 °C, and the acid concentration was 0.5 M, over a period of 180 min. The results are shown in Figure 9. It is possible to observe that the efficiency of Nb and Ta is significantly affected by the solid–liquid ratio. The highest degree of material solubilization was achieved at a ratio of 20 g/L, obtaining recoveries of 89% for Nb and 88% for Ta; while the associated elements, such as Zr, U, and Th, showed values of 3%, 1%, and 8%, respectively, remaining below the 10% threshold. As the S/L ratio increases up to 33 g/L, an increase in the dissolution of U and Th is observed, reaching 27% and 8.5%, respectively, while Nb, Ta, and Zr decrease to 82%, 77%, and 1%. From this solid–liquid ratio onward, a decreasing trend in the recovery of the target metals is observed, along with a slight increase in the solubilization of the other elements. According to Faraji et al.,^51^ this effect translates to a greater availability of C_2_O_4_^2–^ ions, producing higher leaching kinetics, while a reduction increases the viscosity of the solution, hindering the convection and diffusion of the solid material in the leaching solution.

**Figure 9:**
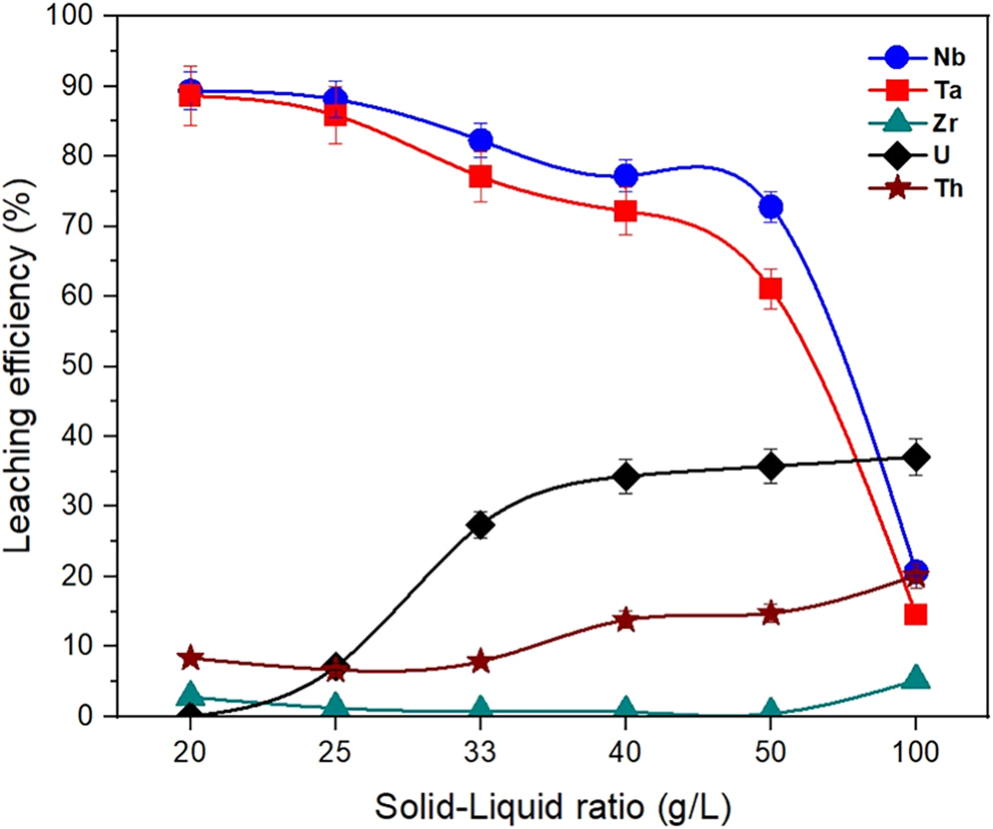
Effect of solid–liquid ratio on niobium and tantalum leaching by thermal treatment (slag/KHSO_4_ (1:3 w/w), 600 °C, 3 h) and leaching (0.5 M, 60 °C, 180 min).

The limited solubility of Th^4+^, Zr^4+^, and U^4+^/UO_2_^2+^ impurities in oxalic medium as the solid–liquid ratio increases is due to the formation of poorly soluble complexes or polymeric species in solution. For example, in the case of thorium, its low dissolution is explained by the formation of complexes with low solubility constants, such as Th­(C_2_O_4_)_2_·6H_2_O (*K*_sp_ ≈ 10^–29^ at 25 °C), and its tendency to precipitate in mildly acidic media.^52^ Similarly, uranium complexes such as UO_2_(C_2_O_4_)·2H_2_O, UO_2_(C_2_O_4_), UO_2_(C_2_O_4_)_2_^2–^, and UO_2_(C_2_O_4_)_2_^4–^ are stable in aqueous media, but their stability and solubility strongly depend on the pH, temperature, and ionic strength of the medium. At high solid–liquid ratios, the concentration of free oxalate in solution decreases, limiting the formation of these soluble uranyl–oxalate complexes and favoring their reprecipitation.^53,54^ Regarding zirconium, it shows a strong tendency to form polymeric hydroxides and hydroxyoxalates, such as Zr­(C_2_O_4_)_2_·*x*H_2_O or Zr­(OH)_2_(C_2_O_4_)­(H_2_O)_2_, with very low solubilities in oxalic medium, particularly in the presence of excess solid.^50^

From the results obtained, increases in the solid–liquid ratio above 33 g/L should be avoided, not only to maintain a moderate efficiency in the recovery of the target metals but also to limit the dissolution of impurities such as Th and Shikika et al.^31^ indicated that above a ratio of 50 g/L, the codissolution of associated metals is significantly restricted. Meanwhile, da Luz reported that at an solid–liquid ratio of 1:30, the leaching efficiencies of Nb and Ta stabilize.^33^ Therefore, as a study strategy, a ratio of 33 g/L was selected.

#### 3.4.2 Effect of Concentration

The effect of H_2_C_2_O_4_ concentration on the recovery of niobium and tantalum was investigated within the range of 0.1 to 2 M at 60 °C for 180 min, with an solid–liquid ratio of 33 g/L. The results, presented in Figure 10, indicate that efficiencies of 82% for niobium and 80% for tantalum can be achieved at a concentration of 0.5 M, together with impurities of 1% Zr, 27% U, and 8% Th. Our hypothesis suggests that increasing the concentration enhances the availability of oxalate ions to form the soluble complexes [NbO­(C_2_O_4_)_3_]^3–^ and [TaO­(C_2_O_4_)_3_]^3–^.^46^ The stability of the formed complexes depends on the acidity region required for Nb (pH 0.5–3) and Ta (pH 4.5–5.5) complexes,^31^ which could explain the difference in the behavior of both elements. This is corroborated by the increase in concentration, as it was observed that above 0.5 M the dissolution efficiencies tend to decrease due to lixiviant saturation and high acidity, which leads to complex imbalance and coprecipitation of complexes, such as metallic Fe oxalates, which may coprecipitate with the target metals.^33^

**Figure 10:**
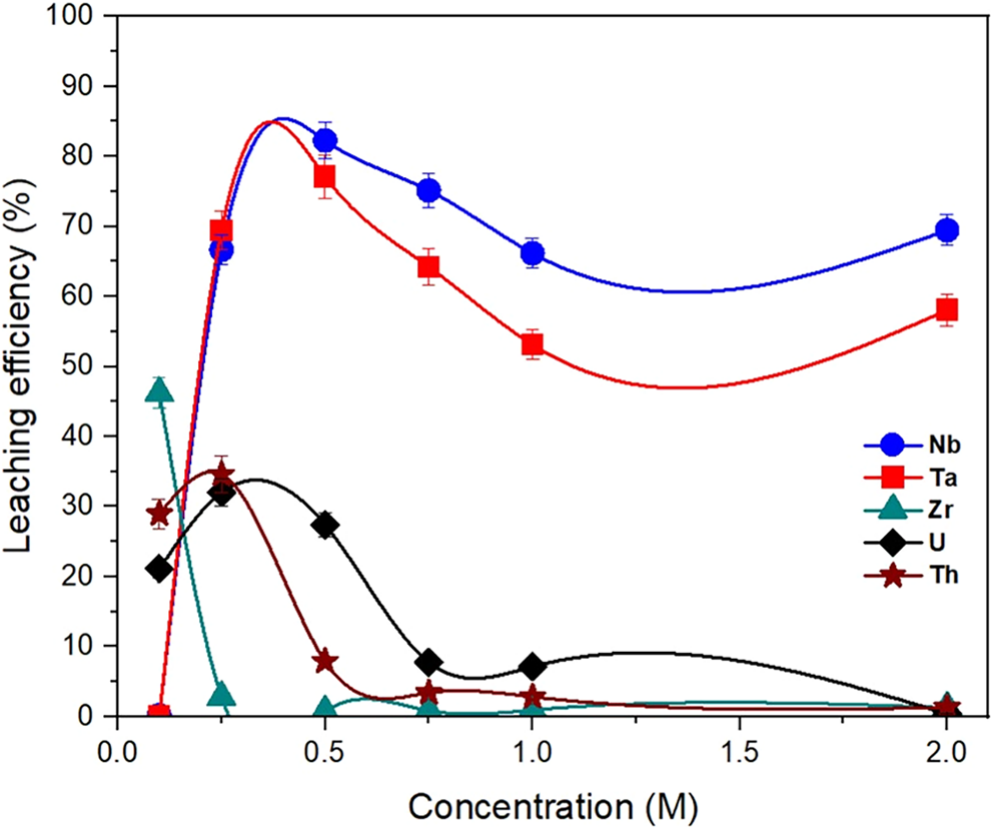
Effect of H_2_C_2_O_4_ concentration on the leaching of niobium and tantalum by thermal treatment (slag/KHSO_4_ (1:3 w/w), 600 °C, 3 h) and leaching (33 g/L, 60 °C, 180 min).

However, the behavior of zirconium at low concentrations (0.1 M) shows an efficiency of 46%, evidencing that the lower the availability of the oxalate ion, the more it remains in solution through hydrolysis processes, expanding its stable solubility region,^39^ and an increase in concentration only leads to the formation of basic oxalates such as Zr­(OH)_2_(C_2_O_4_) or [Zr_2_(C_2_O_4_)_5_(H_2_O)_4_]^2–^, even at moderate oxalate concentrations.^50^ In contrast, Th^4+^ and U^4+^ show analogous extraction behavior: at 0.25 M, the extraction efficiency reaches 34% and 32%, respectively, which may be due to the predominance of slightly soluble intermediate species.^55^ An increase in concentration up to 2 M leads to a decrease in their efficiencies to 0.5% and 1.3% for Th and U, respectively. This behavior may be attributed to the shift in equilibrium toward the formation of soluble anionic complexes in the form of Th­(ox)_4_^4–^ and UO_2_(ox)_2_^2–^.^48,53^ Beyond this point (pH < 2.5), it only leads to the formation of insoluble complexes such as Th­(ox)_2_·6H_2_O_(s)_ and UO_2_(ox)_(s)_.^48^

#### 3.4.3 Effect of Temperature

In previous studies, it was demonstrated that the leaching temperature is not a key variable in the behavior of the material treated after thermal treatment, since this is mainly a salt dissolution process.^24,31,33,39^ The experimental results of this study suggested that the same situation existed in our process. The effect of temperature was evaluated from 25 to 90 °C over 180 min, with an solid–liquid ratio of 33 g/L and a concentration of 0.5 M. Figure 11 shows a slight progression in the leaching of niobium and tantalum with increasing temperature. At 25 °C, the efficiency for both metals is 75%, and a progressive increase in temperature up to 60 °C favors extraction to 82% Nb and 77% Ta. With the increase in temperature up to 90 °C, a slight increase in niobium is observed, reaching 85%, while the efficiency of tantalum decreases with increasing temperature to 65%. According to Anes et al. and da Luz, a positive kinetic effect on niobium efficiency was demonstrated with increasing temperature, whereas the behavior of tantalum remained variable.^33,56^ However, above 60 °C, tantalum recovery decreases, likely due to unfavorable reaction kinetics at higher temperatures, leading to the destabilization of metal complexes and the formation of an oxide layer around the metal. Amer et al.^44^ emphasized that at elevated temperatures, metal oxides such as Nb_2_O_5_ and Ta_2_O_5_ can become more stable, forming layers that inhibit effective leaching.

**Figure 11:**
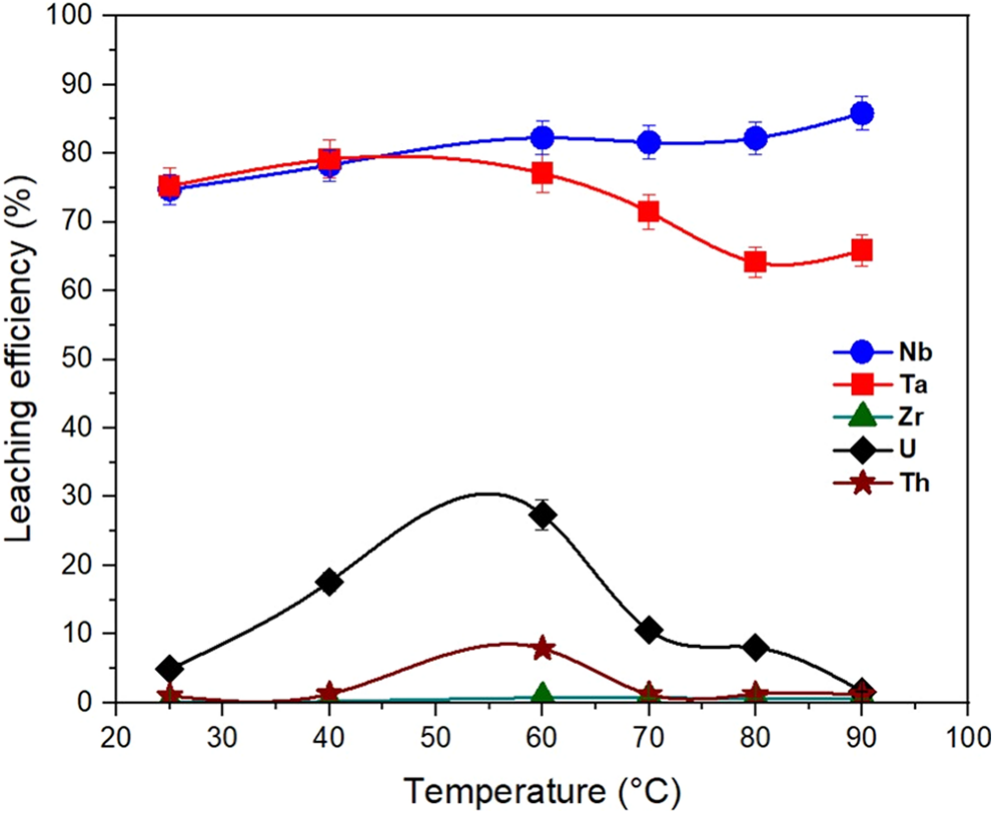
Effect of temperature on the leaching of niobium and tantalum by thermal treatment (slag/KHSO_4_ (1:3 w/w), 600 °C, 3 h) and leaching (33 g/L, 0.5 M, 180 min).

In the leaching of thorium and uranium, it is evident that at 60 °C asymptotic values are reached with increasing temperature. In the case of uranium, an increase in extraction is observed from 5% (25 °C) to 34% (60 °C); this increase in efficiency is possibly due to reoxidation processes that keep it in solution, as shown in eqs 11 and 12.^57^ For thorium, the efficiency at room temperature is 1%, and with the increase in temperature up to 60 °C it reaches a minimum dissolution of 8%, but higher temperatures cause the rapid precipitation of thorium as insoluble thorium oxalate, as shown in eq 13.^52,57^ In the work of Lapidus and Doyle,^48^ it was shown that at 60 °C the maximum dissolution of thorium and uranium is reached, but beyond this temperature the metals are occluded or encapsulated due to the formation of insoluble complexes, explaining the metallic behavior of these elements with increasing temperature.

11 $${\mathrm{UO}}_{2}\left({\mathrm{SO}}_{4}\right)+H_{2}C_{2}O_{4}\to {\mathrm{UO}}_{2}\left(C_{2}O_{4}\right)+H_{2}{\mathrm{SO}}_{4}$$

12 $${\mathrm{UO}}_{2}\left(C_{2}O_{4}\right)+H_{2}{\mathrm{SO}}_{4}\overset{\Delta }{\to }{\mathrm{UO}}_{2}\left({\mathrm{SO}}_{4}\right)+2{\mathrm{CO}}_{2}+2H^{+}$$

13 $$\mathrm{Th}{\left({\mathrm{SO}}_{4}\right)}_{2}+2H_{2}C_{2}O_{4}\overset{\Delta }{\to }{\mathrm{Th}\left(C_{2}O_{4}\right)}_{2}+2H_{2}{\mathrm{SO}}_{4}$$

14 $$\mathrm{Zr}{\left({\mathrm{SO}}_{4}\right)}_{2}+H_{2}C_{2}O_{4}\overset{\Delta }{\to }\mathrm{Zr}{\left(C_{2}O_{4}\right)}_{2}\cdot {nH}_{2}O+2H_{2}{\mathrm{SO}}_{4}$$

However, for zirconium, no appreciable leaching efficiency (<1%) is observed throughout the temperature range studied. This may be due to the fact that after thermal treatment, zirconium remains as ZrO_2_ or in the form of zirconates (ZrSiO_4_), which are highly refractory and have low solubility (Figure 5). Despite the excellent complexing ability of oxalate ions compared to sulfate ions (C_2_O_4_^2–^ > SO_4_^2–^).^58,59^ Studies have shown that the formation of Zr­(C_2_O_4_)_2_ may be dominated by the influence of temperature, which can affect the solubility of the compounds formed or alter the reversibility of the chemical reactions,^51,60^ precipitating as Zr­(C_2_O_4_)_2_·*n*H_2_O eq 14 or as basic oxalate Zr­(OH)_2_(C_2_O_4_),^50,60,61^ thus reinforcing the selectivity of the proposed method.

#### 3.4.4 Effect of Time

Similar to temperature, the literature highlights that time is not a key parameter in the dissolution of the material.^24,31,33^ The effect of time on leaching was evaluated over a range of 60 to 180 min, with a solid/liquid ratio of 33 g/L and a concentration of 0.5 M at 60 °C. The results indicated that increasing the leaching time did not significantly affect the extraction of niobium and tantalum (Figure 12). A period of 60 min was sufficient to obtain leachates with concentrations of 82% niobium and 80% tantalum, values very close to those obtained at 180 min (82% Nb and 77% Ta). However, it can be observed that there is a drastic reduction in the efficiency of radionuclides at shorter reaction times. In the case of uranium, the extraction decreases from 34% (180 min) to 4% (60 min), while for thorium it decreases from 8% (180 min) to 2% (60 min), and zirconium remains below 1% over the entire range of evaluated times.^31,33,42^ These results suggest that shorter times hinder the kinetics of impurity complexation. Considering that the presence of thorium and uranium limits industrial processes, a period of 60 min is appropriate to maximize the extraction of Nb and Ta, producing a Pregnant Leach Solution (PLS) suitable for a potential purification step.

**Figure 12:**
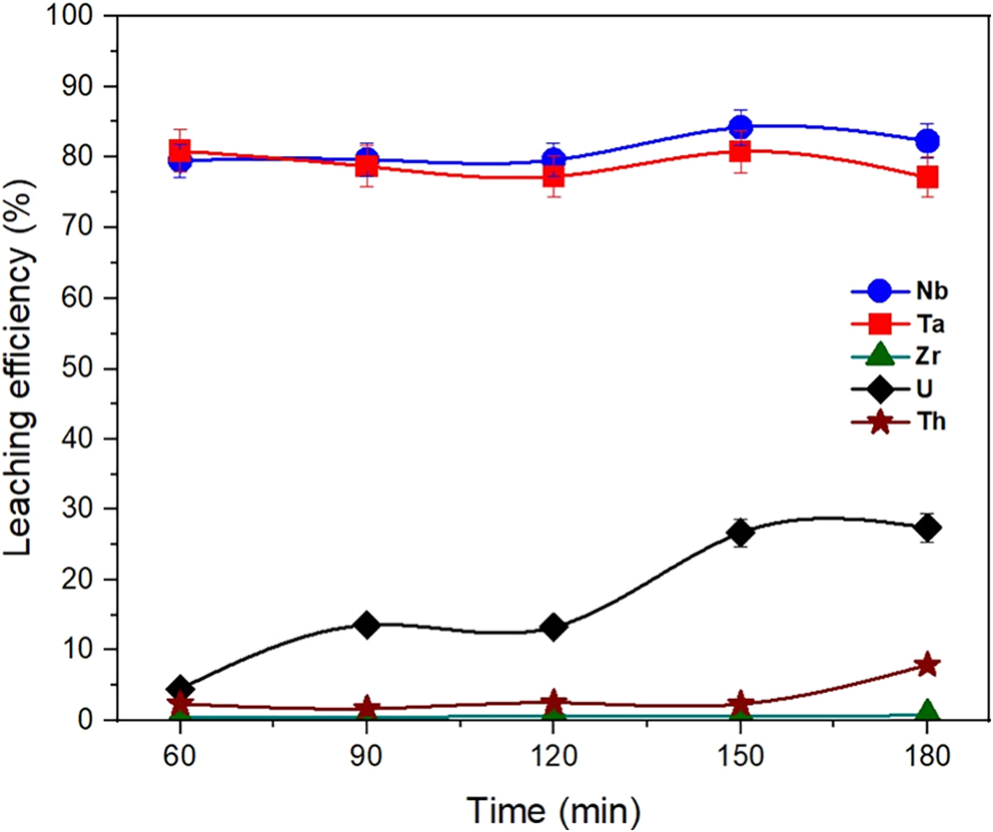
Effect of time on the leaching of niobium and tantalum by thermal treatment (slag/KHSO_4_ (1:3 w/w), 600 °C, 3 h) and leaching (33 g/L, 0.5 M, 60 °C).

### 3.5 Characterization of the Leaching Residue

The chemical characterization of the leaching residue obtained under optimal process conditions is summarized in Table 3. The residue was generated after a thermal treatment stage using KHSO_4_ at a slag/bisulfate ratio of 1/3 at 600 °C for 3 h, followed by aqueous leaching using 0.5 M oxalic acid at a pulp density of 33 g/L and 60 °C for 60 min. The residual contents of Nb and Ta were 1.10 wt % and 0.18 wt % as Nb_2_O_5_ and Ta_2_O_5_, respectively. These values are lower than those in the initial composition and indicate the effective extraction of niobium and tantalum during the roasting-leaching process.

**Table 3:** Elemental Analysis of the Leaching Residue

Oxide	wt %	Oxide	wt %
SiO_2_	35.5	Nb_2_O_5_	1.10
ZrO_2_	15.1	UO_2_	0.54
K_2_O	15.76	TiO_2_	0.46
CaO	12.6	MgO	0.42
SO_4_	2.84	Na_2_O	0.27
ThO_2_	1.79	MnO	0.24
SnO_2_	1.43	BaO	0.19
Fe_2_O_3_	1.37	Ta_2_O_5_	0.18
Al_2_O_3_	1.21	ZnO	0.16
HfO_2_	1.19	Y_2_O_3_	0.12

It can be observed that the concentration of SiO_2_ and ZrO_2_ remains almost constant after acid leaching due to their high chemical stability and refractory nature. Specifically, the low reactivity of zirconium may be associated with its complex hydrolysis behavior, since complete sulfation generally requires highly aggressive conditions and a large excess of sulfuric acid.^39^ In the present study, the decomposition of the material with potassium bisulfate may be governed by two simultaneous mechanisms. The first involves the persistence of refractory zirconia phases^62^ and the limited diffusion of the reagent through silica-covered particles, which restricts zirconium dissolution.^63^ The second mechanism may involve the formation of zirconium sulfate species during roasting, followed by rapid chelation and hydrolysis with hydroxo–oxalate ions during leaching, leading to the formation of poorly soluble zirconium complexes under acidic conditions.^60,62^

While CaO shows a 30% decrease, possibly favored by the reactivity of Ca^2+^ in acidic media (pH < 2). On the other hand, a concomitant increase in K_2_O is observed, which is produced by the addition of KHSO_4_ during the thermal process, resulting in the presence of sulfur in the form of SO_4_. Similarly, the radionuclides remain almost entirely in the residue, although it has been observed that bisulfate has the ability to solubilize these metals in an aqueous medium, as reported in the literature.^21–23^ The combined effect of sulfate ions in solution and oxalates induces the destabilization of soluble complexes and promotes the precipitation of highly insoluble oxalate–sulfate salts, which explains the almost null transfer to the solution and their retention in the leaching residue. This demonstrates the method as a sustainable alternative for producing PLS highly enriched in the target metals, which is necessary for subsequent separation and purification stages.

The mineralogical characterization (Figure 13) reveals the presence of nonreactive crystalline phases such as SiO_2_ (PDF 00–081–1665). The presence of ZrO_2_ (PDF 00–083–0941) evidence the predominance of crystalline zirconia,^62^ together with crystalline zirconium hydroxo–sulfate–oxalate species formed during the extraction process, which may not be completely detected by XRD analysis. Additionally, it is possible to observe that during the process, a peak of hydrated calcium silicate Ca_5_(Si_6_O_17_)·5H_2_O (PDF 00–089–6457) is formed. According to da Luz, the characterization of the oxalic acid leach residue reflected peaks of ZrO_2_ and Ca_6_Si_13_O_12_·(H_2_O), showing consistency with the results obtained in this study.^33^

**Figure 13:**
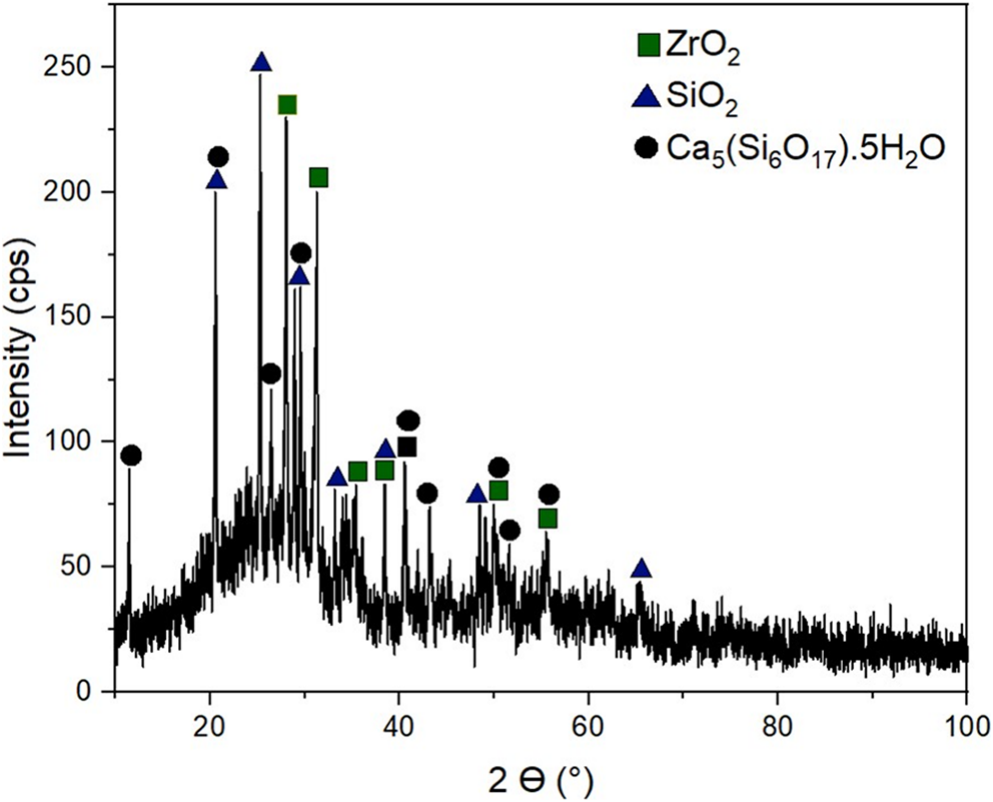
X-ray diffraction (XRD) pattern of the leach residue.

These results indicate that the leaching mechanism is governed by the nature of the phases present in the treated matrix. The absence of noticeable changes in the dominant crystalline phases, as evidenced by X-ray diffraction, suggests that the acid does not induce a generalized surface attack, but rather promotes selective dissolution of Nb and Ta sulfate species formed during roasting.^64^

Such a response is characteristic of a phase-controlled mechanism, in which chemical attack is restricted to more reactive or chemically activated domains, while stable phases, particularly zirconium- and silica-rich phases, remain largely inert due to their high thermodynamic stability.^65,66^ Consequently, leaching proceeds through selective dissolution driven by the differential reactivity of the phases, leading to the enrichment of Nb and Ta in the leachate, while impurities such as Th, U, and Zr are predominantly retained in the solid residue.

### 3.6 Comparison of Oxalic Acid with Other Organic Acids for Nb and Ta

After defining the optimal leaching parameters with H_2_C_2_O_4_, comparative tests were carried out using H_6_C_4_O_5_ (malic acid), H_6_C_4_O_6_ (tartaric acid), H_8_C_6_O_6_ (ascorbic acid), and EDTA.^48^ The results are presented in Figure 14.

**Figure 14:**
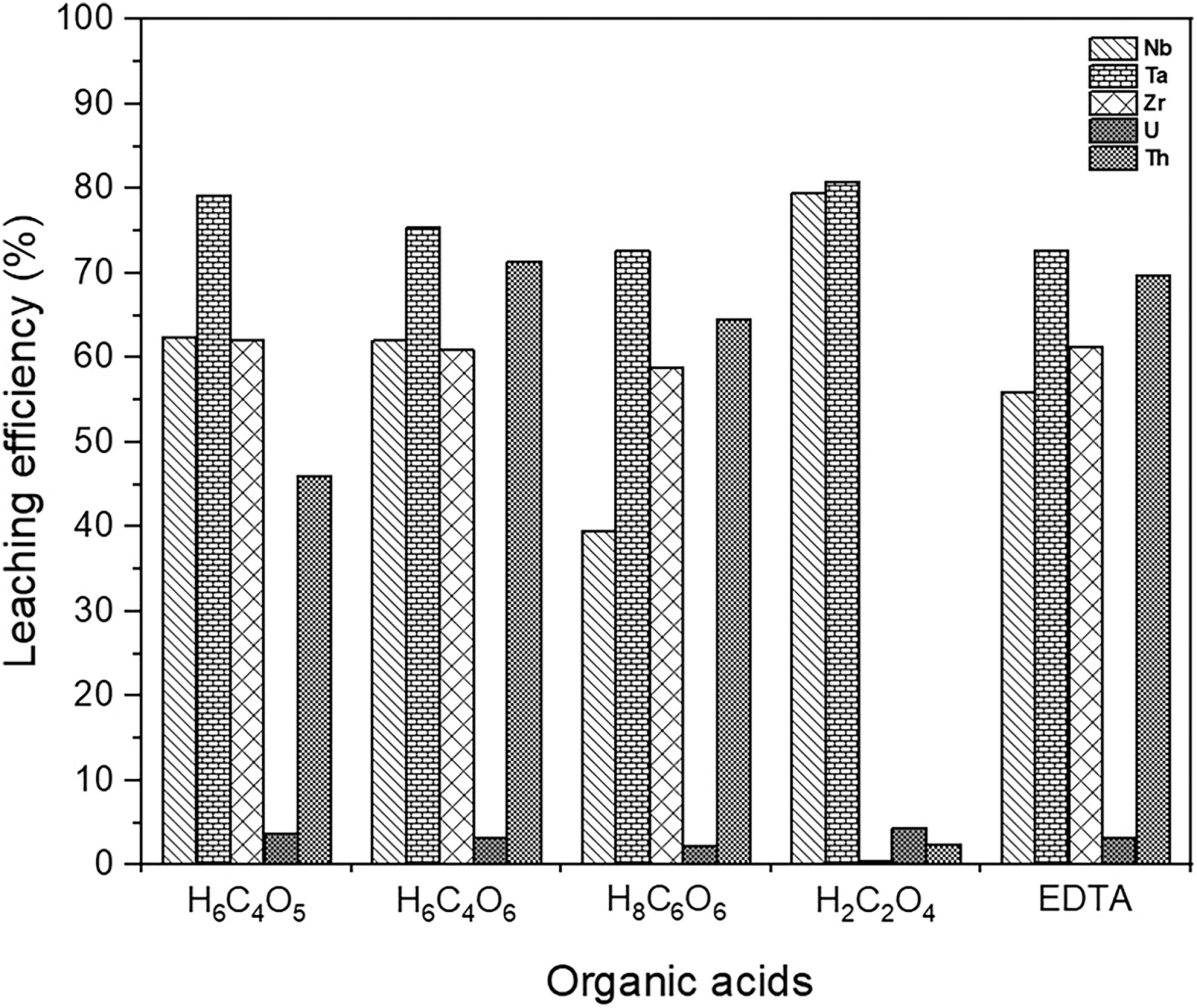
Effect of organic acids on the leaching of niobium and tantalum by thermal treatment (slag/KHSO_4_ (1:3 w/w), 600 °C, 3 h) and leaching (33 g/L, 0.5 M, 60 °C, 60 min).

As shown, oxalic acid exhibited the highest leaching efficiency for Nb and Ta, reaching approximately 82% and 80%, respectively. In comparison, the other organic acids showed lower efficiencies for Nb: 62% for H_6_C_4_O_5_, 62% for H_6_C_4_O_6_, 40% for H_8_C_6_O_6_, and 55% for EDTA. A similar trend was observed for Ta, with values of 78% (H_6_C_4_O_5_), 75% (H_6_C_4_O_6_), 72% (H_8_C_6_O_6_), and 73% (EDTA), all lower than those obtained with oxalic acid.

This aligns with what was predicted by Lapidus and Doyle.,^48^ who showed that EDTA and citrate ligands have higher affinity for thorium and uranium. This has also been evidenced by Choppin et al.,^67^ who showed that the citrate–thorium complex in solution is stable due to the chelating effect, ligand geometry, and high stability constant (β^1^ ≈ 10–12 and log β^2^ ≈ 21–26), which is higher than the stability constant of thorium–oxalate complexes (β^1^ ≈ 5–7 and log β_3_ ≈ 14–16),^49^ thus demonstrating the high leaching efficiencies of Th and U by multidentate ligands.

Furthermore, another reason for the superiority of H_2_C_2_O_4_ in the leaching of niobium and tantalum, compared to other organic complexing agents, lies in its chemical structure and high ionization constants (*k*_a1_ = 5.6 × 10^–2^ and *k*_a2_ = 5.4 × 10^–5^),^47^ which allow the formation of stable complexes. According to da Luz, four organic acids (citric, oxalic, acetic, and tartaric acids) were evaluated against three inorganic acids for leaching. In this context, the high complexation efficiency of oxalic acid was highlighted, achieving more than 90% efficiency for Nb and nearly 50% for Ta.^33^

Considering the process developed in this study, the objective is to recover niobium and tantalum from tin slag using an organic acid as a leaching agent. The use of H_2_C_2_O_4_ not only minimizes the dissolution of impurities but also reduces the need for processes involving inorganic acids, such as HF, thereby contributing to the reduction of environmental impacts. This approach is crucial for the development of more efficient and environmentally responsible hydrometallurgical routes for the extraction of refractory metals.

### 3.7 Process Feasibility

To evaluate the feasibility of the process, a comprehensive assessment was conducted, considering both the mass balance and the economic aspects associated with the profitability of the developed methodology.

#### 3.7.1 Partial Mass Balance

The flow diagram presented in Figure 15 was developed based on the processing of 1000 kg of tin slag, with an initial composition of 5.5% Nb_2_O_5_, 0.6% Ta_2_O_5_, 15.5% ZrO_2_, 0.6% UO_2_, and 2.1% ThO_2_, corresponding to 38.7 kg, 5.3 kg, 114.8, 5.0, and 18.5 kg, respectively. The process begins with acid roasting using KHSO_4_ under conditions of a slag-to-KHSO_4_ ratio of 1:3 (w/w) at 600 °C for 3 h. Subsequently, the roasted material was subjected to leaching with 0.5 M H_2_C_2_O_4_, using a solid-to-liquid ratio of 33 g/L at 60 °C for 60 min.

**Figure 15:**
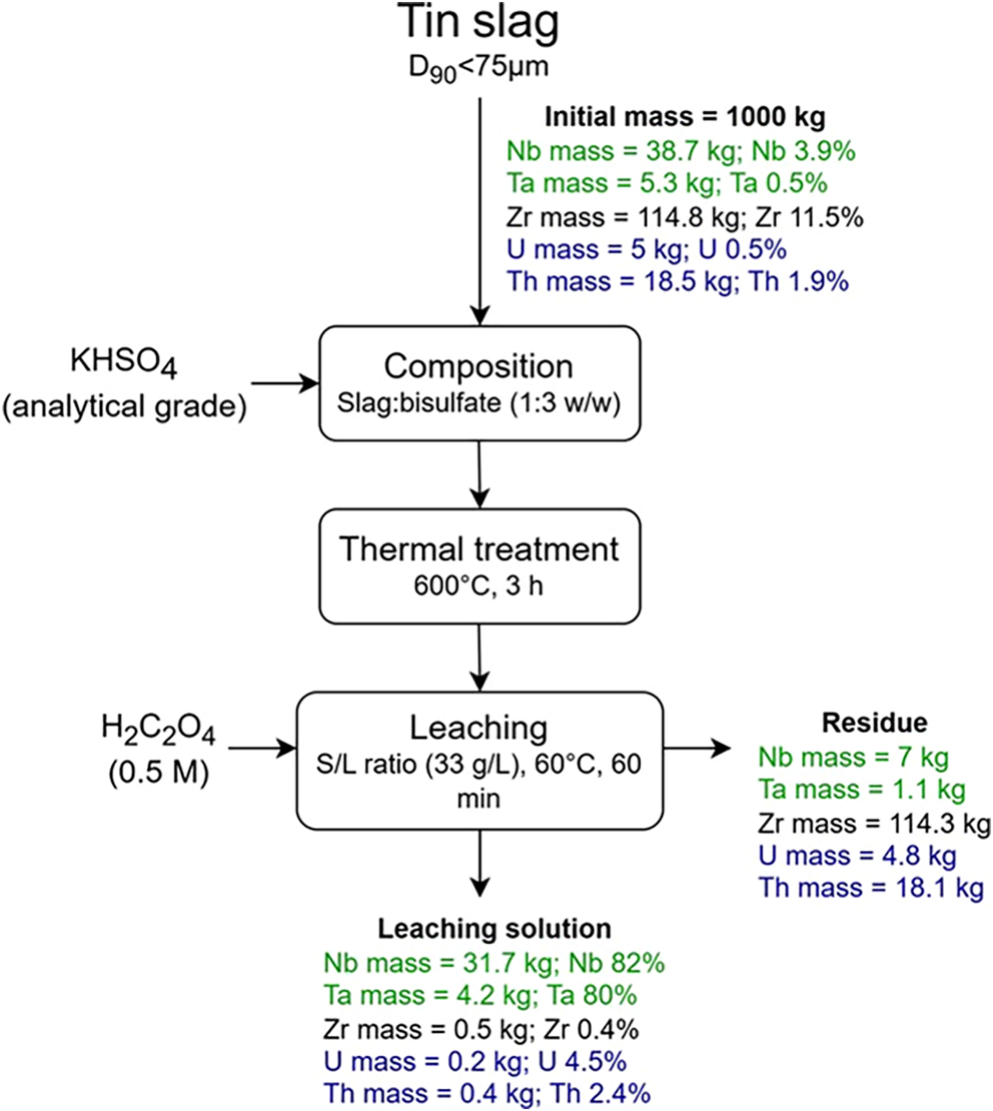
Partial mass balance for 1000 kg of tin slag through the proposed pyrometallurgical–hydrometallurgical route.

Through the proposed methodology, it can be observed that two streams were generated after the leaching stage: the leachate solution and the solid residue. The leachate solution contained 31.7 kg of Nb (82%) and 4.2 kg of Ta (80%), while Zr, U, and Th were present in amounts lower than 1 kg. According to international market prices, Nb (ingot, minimum purity 99.8%, Rotterdam warehouse) can reach approximately 95 USD/kg, whereas Ta (minimum purity 99.8%, Rotterdam) is priced at around 305 USD/kg.^68^ These values represent an economic contribution of approximately 400 USD/kg for the target metals present in the leach liquor.

On the other hand, the leaching residue obtained is emerging as a potential resource for the extraction of metals with more advanced interest in industry. This is evident, as a large amount of radioactive material such as uranium and thorium is observed, representing a total mass of 4.8 and 18.1 kg, respectively. Demonstrating the need for further studies focused on the recovery of this type of metals. Likewise, it is important to highlight that there is a considerable amount of Nb (7 kg) and Ta (1.1 kg) yet to be recovered; as well as strategic elements such as zirconium, hafnium, and titanium, which increase the added value of the material, and are considered critical materials by major economies.^69,70^

#### 3.7.2 Economic Feasibility

The economic evaluation of the process is important, as it allows us to predict the economic impact of the developed route. If we consider the reagents involved, the energy cost, and the potential value of niobium and tantalum obtained in the leaching solution, whose results are shown in Table S1 (Supporting Information). From these data, we can deduce that the cost of oxalic acid (22%) and electricity (1%) are not significant in the overall balance. However, bisulfate represents 77% of the total process cost, which could reduce the economic contribution derived from the target metals. Although the reagent tends to represent the largest economic fraction, its use is justified by its ability to promote selective extraction of the target metals over matrix impurities, such as radionuclides.

Taking as reference the costs presented in Table S1, it is possible to establish comparisons with other methods reported in the literature. For example, roasting with sulfuric acid is usually more economical, since the acid acts as a universal solvent and its cost is 99% lower than bisulfate (0.098 USD/kg).^24^ However, this method may be hindered because it promotes the nonselective dissolution of metals, increasing operating costs in subsequent separation and purification stages. Similarly, the use of bases in thermal treatment stages has been proposed as a more eco-friendly alternative and is 98% lower than bisulfate (0.6 USD/kg); they have a dissolution effect similar to acidic methods, resulting in partial or total dissolution of the elements in solution.^31,33^ So far, studies focused on the extraction of niobium and tantalum are only aimed at maximizing their recovery, omitting the presence of impurities. Thus, this study seeks a balance between maximizing extraction and reducing impurities. Opening new avenues of study, focused on producing clean PLS to facilitate the subsequent operation of the processes.

## 4 Conclusions

The present study demonstrates the feasibility of the selective extraction of niobium and tantalum from tin slag matrices without the presence of HF or its derivatives. The developed methodology includes a thermal treatment step of the slag with KHSO_4_ at 600 °C, using a slag-KHSO_4_ ratio of 1:3 (w/w) for 3 h to ensure the formation of soluble sulfate compounds, necessary for the subsequent leaching stage. During this stage, the experimental conditions were predicted through thermodynamic modeling, evidencing an optimal practice of the process. Leaching in an oxalic medium, with a solid-to-liquid ratio of 33 g/L, 0.5 M, at 60 °C for 60 min, showed that the solution contained more than 80% of niobium and tantalum, with impurities such as silicon, zirconium, thorium, and uranium predominating in the leaching residue after evaluation by WEDXRF. The results indicate a viable methodology for the selective leaching of niobium and tantalum without HF, with low levels of thorium and uranium, which are common challenges in secondary residue metallurgy. Future research could focus on the clean process for further purification of the obtained leachate.

## Supplementary Material



## Notes

Particle size distribution curve in the original tin slag and particle size analysis of the comminuted slag (PDF)
