# Supplementary material for: Selective Extraction Process of Niobium and Tantalum from Tin Slag Derived from the Thermal Processing of Cassiterite
Source: ACS Omega. 2026 Jul 8;11(28):41794–808. doi: 10.1021/acsomega.6c01589 (PMC13392889; doi:10.1021/acsomega.6c01589)
Supplement: Supplementary file 1 [file ao6c01589_si_001.pdf]

Supplementary Material for *ACS Omega*

Selective extraction process of niobium and tantalum from tin slag derived from the thermal processing of cassiterite

Darwin Michell Cheje Machaca[a,b*]; Rosario Belen Juyo Salazar[a,b*]; Susan Sofia Flores-Calla[c]; Amilton Barbosa Botelho Junior[d]; Denise Crocce Romano Espinosa[a]; Jorge Alberto Soares Tenório[a]

[a]Department of Chemical Engineering, Polytechnical School, University of Sao Paulo, São Paulo, SP 05508-080, Brazil.

[b]Universidad Nacional de San Agustín de Arequipa, Arequipa 04000, Peru

[c]Catholic University of Santa Maria, Arequipa, Perú

[d]Department of Chemical Engineering, Norwegian University of Science and Technology, Trondheim 7491, Norway

*Corresponding author(s): Darwin Michell Cheje Machaca, E-mail(s): dcheje12@usp.br

Rosario Belen Juyo Salazar, E-mail(s): rjuyo@usp.br

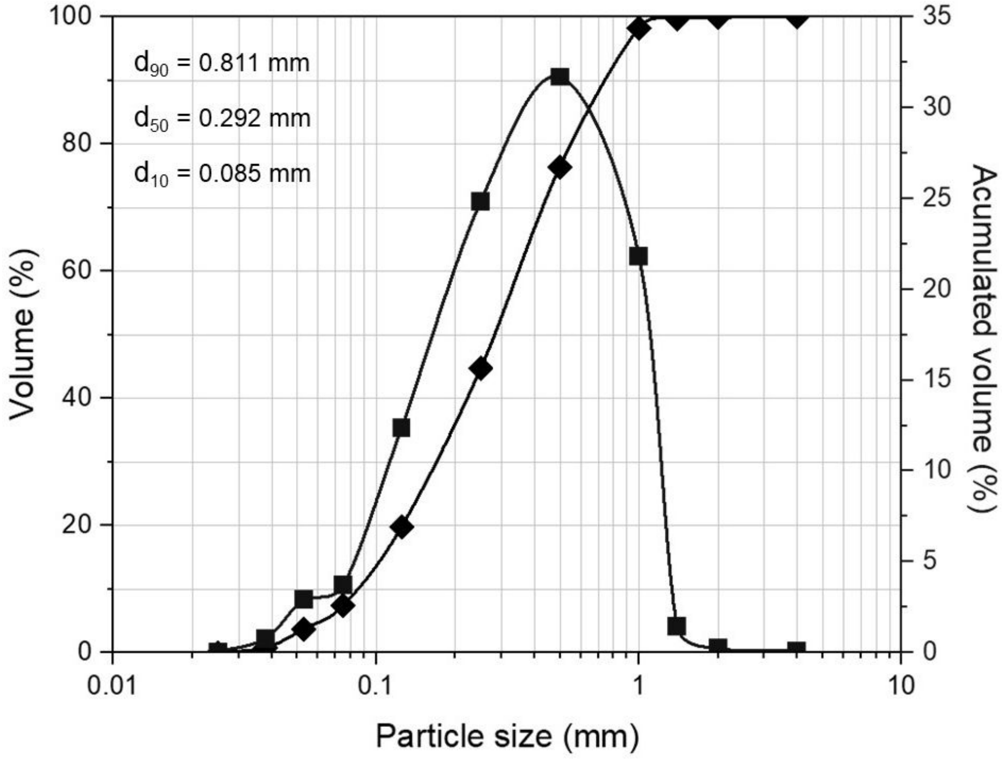

Figure S1 - Particle size distribution curve in the original tin slag (performed using a set of sieves arranged in a mechanical shaker (GLASSLAB 12499).

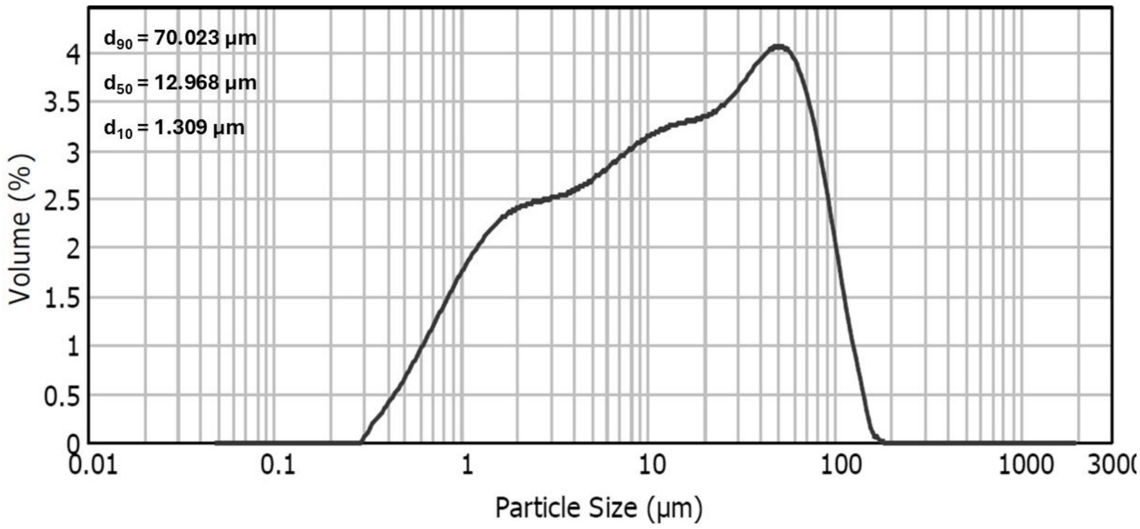

Figure S2 - Particle size analysis of the comminuted slag (performed using the MasterSizer 2000 instrument)

Table S1 - Influence of reagent cost and energy consumption

**Material**	**Cost (USD/kg)**
$KHSO_4$	27.52
$H_2C_2O_4$	7.98
Nb_2O_5	95
Ta_2O_5	305
Electricity (kWh)	0.12*

Note: * = value only in USD (consumption between 5–10 kWh)
